# Liquid Droplet Aging and Seeded Fibril Formation of the Cytotoxic Granule Associated RNA Binding Protein TIA1 Low Complexity Domain

**DOI:** 10.1021/jacs.2c08596

**Published:** 2023-01-13

**Authors:** Yuuki Wittmer, Khaled M. Jami, Rachelle K. Stowell, Truc Le, Ivan Hung, Dylan T. Murray

**Affiliations:** † Department of Chemistry, University of California Davis, Davis, California 95616, United States; ‡ National High Magnetic Field Laboratory, Tallahassee, Florida 32310, United States

## Abstract

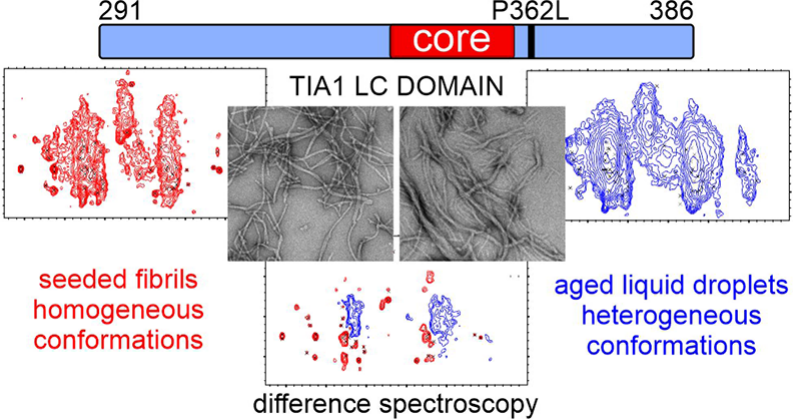

Protein domains biased toward a few amino acid types are vital for the formation of biomolecular condensates in living cells. These membraneless compartments are formed by molecules exhibiting a range of molecular motions and structural order. Missense mutations increase condensate persistence lifetimes or structural order, properties that are thought to underlie pathological protein aggregation. In the context of stress granules associated with neurodegenerative diseases, this process involves the rigidification of protein liquid droplets into β-strand rich protein fibrils. Here, we characterize the molecular mechanism underlying the rigidification of liquid droplets for the low complexity domain of the Cytotoxic granule associated RNA binding protein TIA1 (TIA1) stress granule protein and the influence of a disease mutation linked to neurodegenerative diseases. A seeding procedure and solid state nuclear magnetic resonance measurements show that the low complexity domain converges on a β-strand rich fibril conformation composed of 21% of the sequence. Additional solid state nuclear magnetic resonance measurements and difference spectroscopy show that aged liquid droplets of wild type and a proline-to-leucine mutant low complexity domain are composed of fibril assemblies that are conformationally heterogeneous and structurally distinct from the seeded fibril preparation. Regarding low complexity domains, our data support the functional template-driven formation of conformationally homogeneous structures, that rigidification of liquid droplets into conformationally heterogenous structures promotes pathological interactions, and that the effect of disease mutations is more nuanced than increasing thermodynamic stability or increasing β-strand structure content.

## Introduction

Granular condensates of proteins and nucleic acids are integral to RNA metabolism. These membraneless organelles facilitate RNA processing and transport, and control proteostasis during cellular stress.^1,2^ Macroscopic, fluorescence-based measurements on cultured cells show that these RNA granules contain a heterogenous collection of molecules undergoing varying levels of molecular motion.^3^ Experiments using purified proteins reveal that the condensation of homogeneous solutions of specific proteins into liquid droplet structures reproduces the macroscopic behavior of *in vivo* RNA granules.^4,5^ A variety of weak, multivalent interactions facilitate this behavior, which is dictated by the properties of the specific proteins involved.^6^

A low complexity (LC) domain is a protein segment that is biased toward a subset of the 20 amino acid types typically used for natural protein synthesis.^7^ LC domains are well represented in proteins that form a variety of macroscopic assemblies in living cells^8^ and are of significant interest in the context of biomolecular condensation. These sequences inherently contain the multivalent property required for liquid droplet formation^9^ and can assemble into more rigid amyloid-like fibrils functionally and pathologically.^10,11^ Liquid droplets are normally characterized by significant molecular motion and disorder.^4^ The aging of the liquid droplets, a process where the droplets adopt a more rigid structure with potentially more homogeneous molecular conformations, is particularly pertinent for understanding how RNA granules fail to disassemble, facilitating the buildup of protein aggregates.^12^

The misassembly of LC domain proteins is intricately linked to neurodegenerative diseases. Amyotrophic lateral sclerosis (ALS) is a progressive neurodegenerative disease of the spinal cord and brain that results in the loss of muscle movement.^13^ Frontal temporal dementia (FTD) is the second most common form of dementia after Alzheimer’s disease.^14^ These two diseases have many genetic links to LC domain proteins involved in the formation of RNA granules.^15^ The RNA-binding protein TIA1 (Cytotoxic granule associated RNA binding protein TIA1) has ALS and FTD missense mutations clustered in a C-terminal LC domain.^16^ While the buildup of TIA1-rich inclusions is not typical of ALS and FTD pathology, TIA1 disease mutations result in the persistence and rigidification of stress-related RNA granules and are associated with the pathological deposition of other LC domain proteins.^16^

Functional activity of the TIA1 protein includes the regulation of RNA splicing and translation.^17^ The TIA1 LC domain is a 96 amino acid sequence biased toward Gln, Gly, Tyr, and Pro residues that assembles into amyloid-like fibrils and liquid droplets *in vitro*.^16,18,19^ Organized and reversible fibrillar self-assembly is possibly a functional activity for the domain.^20^ Yet, despite significant interest in TIA1-mediated condensation processes, there are few structural characterizations of TIA1 fibril formation. The P362L LC domain mutation delays the disassembly of functional full-length TIA1 assemblies.^18^ Several Pro-to-hydrophobic mutations in the TIA1 LC domain lead to increased aggregation rates for the protein and suggest an antipathogenic role for Pro residues.^18^ However, the residue-specific effects of LC domain mutations on the rigidification of TIA1 liquid droplets remain uncharacterized at high resolution. The biological function and pathogenicity of TIA1 involves LC domain self-assembly processes for which the molecular mechanisms remain unknown.^20^

Here, we report the results from solid state nuclear magnetic resonance (NMR) measurements, electron and bright field microscopy imaging, and fluorescence assays that characterize condensed states of wild-type and P362L mutant forms of the TIA1 LC domain. High-resolution measurements of seeded fibrils report on the precise location of the most fibril-prone region of the protein. Analysis of aged liquid droplets provide insights into the structural changes underlying liquid droplet rigidification and how an ALS and FTD mutation affects the process. Our results provide a basis for understanding the sequence context of disease mutations in the TIA1 LC domain as it relates liquid droplet aging and contribute to our understanding of the broader conformational space sampled by LC domains during proper biological function and disease pathology.

## Experimental Section

### Protein Expression, Purification, and Site-Directed Mutagenesis

His-tagged wild-type and P362L mutant TIA1 LC domains (residues I291–Q386) were recombinantly expressed in *E. coli* and purified under denaturing conditions using Ni^2+^ affinity chromatography. The P362L mutant was obtained using standard polymerase chain reaction-based methods. The complete details of these procedures are provided in the Supporting Information.

### Preparation of TIA1 LC Domain Seeds

TIA1 LC domain protein in 500 mM sodium chloride, 6 M urea, ∼60 mM imidazole, and 20 mM 4-(2-hydroxyethyl)-1-piperazineethanesulfonic acid (HEPES), pH 7.5 at 355 μM, was buffer-exchanged into 20 mM Tris (2-amino-2-(hydroxymethyl)propane-1,3-diol), 200 mM sodium chloride, pH 7.5, using a 500 μL centrifugal concentrating device (Amicon Ultra, 3 kDa). The protein was diluted to 70 μM in 20 mM Tris, 40 mM sodium chloride, pH 7.5, tip-sonicated, and incubated overnight at ∼20 °C. The protein was next diluted to 18 μM in 20 mM HEPES, pH 7.5, and tip-sonicated to make seeds. A solution of 70 μM pure TIA1 LC domain was dialyzed into 20 mM HEPES, pH 7.5, overnight and centrifuged prior to adding the seeds in a 1% mass ratio. The mixture was incubated with rotation at ∼20 °C for 7 d and tip-sonicated twice during the incubation. Next, the solution was diluted to 17 μM in 20 mM HEPES, pH 7.5, and tip-sonicated. This solution was added at 5% by mass ratio to a centrifuged solution of pure TIA1 LC domain dialyzed into 20 mM HEPES, pH 7.5. The solution was then stored at 4 °C for 25 d before warming to ∼20 °C and diluting to half with a centrifuged 12 μM solution of pure TIA1 LC domain dialyzed into 20 mM HEPES, pH 7.5. The dilution was tip-sonicated before adding to a larger volume of the 12 μM protein at a 5% mass ratio. The protein was incubated quiescently for 13 d. Aliquots of the fibrils were prepared and frozen at −80 °C for later use as seeds. Tip-sonication was performed using a Branson 250 Sonifier equipped with a 1/8-inch microtip operated at 10% power for 1 min total on time in cycles of 0.1 s on and 1 s off. All centrifguation steps were 20,000*g* for 20 min at ∼20 °C.

### NMR Sample Seeding

Pure ^13^C and ^15^N labeled TIA1 LC domain protein was dialyzed from denaturing conditions into 20 mM HEPES, pH 7.5, overnight at a concentration of 180 μM in 6 mL volume and centrifuged. TIA1 LC domain seeds amounting to 1% by mass of the isotopically labeled material were removed from the −80 °C storage, warmed to ∼20 °C, tip-sonicated, and added to the dialyzed TIA1 LC domain protein. The solution was mixed with a pipette and incubated quiescently at ∼20 °C for 7 d. These isotopically labeled fibrils were diluted to 90 μM and tip-sonicated to form seeds. The seeds were added at a 2% mass ratio to centrifuged pure ^13^C and ^15^N labeled TIA1 LC domain dialyzed into 20 mM HEPES, pH 7.5 (180 μM, 6.5 mL). The solution was mixed with a pipette and incubated quiescently for 1 d. The presence of visually homogenous fibrils and the absence of amorphous aggregates was confirmed by negative stain transmission electron microscopy (TEM). Tip-sonication was performed using a Branson 250 Sonifier equipped with a 1/8-inch microtip operated at 10% power for 1 min total on time in cycles of 0.1 s on and 1 s off. All centrifguation steps were 20,000*g* for 20 min at ∼20 °C.

### TIA1 LC Domain Liquid Droplet Formation

For both wild-type and P362L mutant TIA1 LC domain, a total of 15 mg purified protein was diluted to 83 μM using 500 mM sodium chloride, 6 M urea, 200 mM imidazole, and 20 mM HEPES, pH 7.5, and dialyzed against 1 L of 150 mM sodium chloride and 20 mM HEPES, pH 7.5. 65 μL aliquots were taken out of the dialysis tubing after gently pipetting to homogenize and mix the sample, at 90 min, 3 h, and 4 h for fluorescence measurements and microscopy imaging. The protein was transferred from the dialysis bag after 4 h to a 50 mL conical tube and incubated on the benchtop at room temperature to age quiescently. Additional 65 μL aliquots were removed for analysis after gently pipetting to homogenize the sample at 24 h, 48 h, and 1 week. The TIA1 LC domain aged liquid droplet samples were harvested for the NMR measurements by centrifugation after 9 d for the wild-type and at 14 d for the P362L mutant.

### Microscopy

Details of the bright field, TEM, atomic force microscopy, and confocal fluorescence microscopy are provided in the Supporting Information.

### ThT and Intrinsic Fluorescence Assays

Trp fluorescence was measured on neat protein solutions. Thioflavin-T (ThT) fluorescence was measured on samples containing equal parts neat protein solution and 40 μM ThT. The reported values are the average of three measurements with an uncertainty of ±1 standard deviation. Bound ThT was quantified by centrifuging the samples at 233,000*g* and 12 °C for 1 h. The ratio of the 412 nm absorbance of the supernatant to that of a 20 μM ThT control sample in identical buffer conditions was used to determine the amount of bound ThT in the aged liquid droplet samples. Additional details are in the Supporting Information.

### Solid State NMR Data Collection

Seeded TIA1 LC domain fibrils were pelleted at 30,000*g* at 25 °C for 30 min and aged liquid droplet wild-type and P362L mutant TIA1 LC domain were harvested at 233,000*g* at 12 °C for ∼20 h. For all samples, hydrated pellets containing 10–15 mg of TIA1 LC domain protein were transferred into 3.2 mm thin-walled pencil-style zirconia rotors (revolution NMR) with a spatula and packed by centrifugation. To compact the samples, the seeded TIA1 LC domain sample was centrifuged at 25,000*g* for ∼30 h at 8 °C and the aged liquid droplet samples were centrifuged at 25,000*g* for 20 min at 12 °C. The rotor drive tip and top cap were sealed with cyanoacrylate gel. Based on average protein density (1.35 g/cm^3^) and the volume of the NMR rotor (36 μL), the hydrated samples are approximately 30% protein. Residual soluble protein concentration was determined after the initial centrifugation step using absorbance measurements at 280 nm recorded with a 1 mm pathlength and a calculated extinction coefficient of 46,870 M^–1^ cm^–1^ (ProtParam^21^).

Experiments were performed on 18.8 T magnets at the UC Davis NMR Campus Core Facility (Davis, California) and the National High Magnetic Field Laboratory (Tallahassee, Florida). BlackFox NMR and Low-E triple resonance 3.2 mm MAS probes were used for all experiments. A table of data acquisition parameters is provided in Table S1. Unless otherwise specified, the sample temperature was ∼10 °C. For all cross-polarization experiments, 83.3 kHz ^1^H decoupling was used. SPINAL-64 was used for the direct and indirect acquisition periods, and CW was used for the ^15^N–^13^C cross-polarization steps. For the INEPT experiments, 10 kHz WALTZ-16 ^1^H decoupling was used. The observed chemical shifts were externally referenced to the DSS scale with the ^13^C downfield peak of Adamantane at 40.48 ppm using a dehydrated sample of 1–^13^C labeled Ala powder. ^13^C *T*_2_ measurements were made by inserting a variable length spin-echo period immediately after the cross-polarization step in a ^1^H–^13^C 1D experiment. ^15^N *T*_2_ measurements were made by inserting a variable length spin-echo period between the ^1^H–^15^N and ^15^N–^13^CA cross-polarization steps in a 1D NCA experiment. For site-resolved ^15^N *T*_2_ measurements, a 2D experiment was used. Echo periods were varied up to 10.2 ms for ^13^C and 15.2 ms for ^15^N and utilized high power 83.3 kHz ^1^H decoupling. The integrated 1D signal intensity (non-glycyl CA for ^13^C) was fit to single exponentials in TopSpin 3.6 software. The *T*_2_ values from the 2D spectra were extracted using NMRPipe and fit to single exponentials using Python scripts. For all spectra, carbon–carbon correlation spectra were plotted with contours increasing by a factor of 1.4. Nitrogen–carbon correlation spectra were plotted with contours increasing by a factor of 1.25.

### Residue-Specific Assignments

Chemical shift peak tables representing NCACX, NCOCX, and CANCO chemical shift correlations were constructed from the 3D cross-polarization-based data sets^22^ recorded on the seeded TIA1 LC domain fibril sample. A 2D TEDOR-NCACX experiment^23^ was used to identify the NCACX signals for proline residues. Signal assignment was achieved using the MCASSSIGN algorithm^24^ with a procedure that generally followed our previous assignments for LC domain samples.^25−28^ The input signal tables are presented in Tables S2–S4 and do not include weak signals, such as those from the Gly residues highlighted in Figures S1A and S4, that do not have matching signals in the 3D NCOCX spectrum. These signals account for much of the broad signal intensity observed in the 2D NCACX and NCOCX spectra in Figure 2B,C. Due to significant overlap in the CB/CG region corresponding to residues such as Arg, His, Ile, Gln, Glu, Met, Trp, Tyr, Phe, and Leu, signals in this region were given generous uncertainties and allowed to be assigned to multiple residues in initial calculations. The MCASSIGN algorithm was run twice, each round consisting of 50 independent calculations with 20 steps and 10^7^ iterations per step. The GOOD, BAD, EDGE, and USED weights were ramped from (0–10), (10–60), (0–8), and (0–6) during the annealing calculation, and a different random number seed was used for each calculation. After the first round of calculations, signals were assigned for residues T347–N357 in 100% of the calculations. These assignments were fixed for a second round of calculations, which resulted in the same signals being assigned to residues A338–Q346 in 100% of the calculations. Subsequent calculations failed to converge on a unique result due to the large CB/CG uncertainties assigned for some signals and the pseudo-repetitive nature of the TIA1 LC domain sequence. The calculation run with the only His-tag as the input sequence resulted in no signals being assigned consistently to any stretch of residues greater than three, ruling out the possibility of the His-tag being significantly ordered in the fibril structure. A calculation run such that signals could not be assigned to residues A338–N357 resulted in no significant assignment of signals outside this region, ruling out the possibility that some of the chemical shifts derived from multiple residues in similar chemical environments and the possibility of a longer stretch of the TIA1 LC domain immobilized in the fibril core. Finally, a calculation run on the complete TIA1 LC domain with the NCOCX signals assigned to A338–N357 omitted but complete signal tables for the NCACX and CANCO data did not result in any significant assignments, ruling out the possibility of two different strongly ordered conformations for residues A338–N357. There are two sets of observed Thr signals that only differ significantly in their CG2 chemical shifts. The assignment process was unable to distinguish these sites due to their identical CA and CB chemical shifts and nearly identical amide N chemical shifts. Neither of these signals was ever consistently assigned to the very C-terminal Thr site or the two Thr sites in the His-tag.

## Results

### TIA1 LC Domain Fibril Seeding

**Figure 1:**
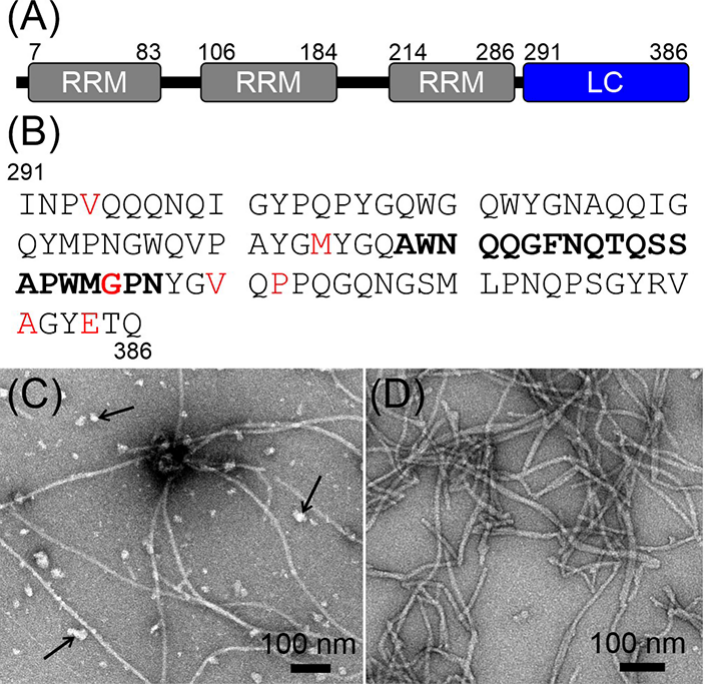
TIA1 Domain Structure and Fibril Seeding. (A) The TIA1 protein is composed of three RRM that precede a 96-residue LC domain. (B) TIA1 LC domain primary sequence in the single-letter amino acid code. The locations of missense mutations linked to neurodegenerative diseases are highlighted in red. The segment that forms the core of the seeded fibrils identified in this work is in bold letters. (C) Negatively stained TEM image of TIA1 LC domain fibrils prior to seeding. Arrows point to amorphous aggregates that form concomitantly with the fibrils. (D) A negatively stained TEM image of the seeded TIA1 LC domain fibrils shows a reduced number of amorphous aggregates.

Figure 1A shows the domain structure of the wild-type full-length TIA1 protein. Three N-terminal RNA-binding motifs (RRM, RNA-recognition motif) are followed by a 96-residue LC domain. The primary sequence of the TIA1 LC domain shown in Figure 1B reveals that the LC domain is biased toward a subset of the 20 most common amino acids and is dominated by 22% Gln, 16% Gly, 12% Pro, 9% Tyr, and 9% Asn. These residues are well distributed across the LC domain. LC domain mutations associated with neurodegenerative disease are colored red in Figure 1B.^16^ These mutations are dispersed along the length of the TIA1 LC domain rather than localized to a specific region of the primary sequence. Ala, Ile, Leu, Met, Val, Trp, Ser, and Thr residues are less common in the TIA1 LC domain. Ala and Val residues are found throughout the LC domain, and Trp is spread along the first two-thirds of the LC domain. The Ile residues are confined to the N-terminal region, and Ser and Thr residues are found in the C-terminal region. The distribution of the less common amino acids makes them highly informative reporters on structure and dynamics in the LC domain.

A seeding procedure like the one used for the β-amyloid peptide^29^ produced homogenous preparations of TIA1 LC domain fibrils. Pure TIA1 LC domain protein was allowed to aggregate at near-neutral pH and moderate ionic strength. Then, the protein was transferred to a low ionic strength solution and sonicated to produce seeds. The seeds were then added to soluble TIA1 LC domain protein at near-neutral pH and low ionic strength, and templated growth was allowed to proceed. The process was repeated sequentially, using the fibrils resulting from templated growth as seeds in the subsequent round, a total of five times with seed to soluble protein mass ratios between 1 and 5%. The negatively stained TEM image in Figure 1C shows that after extensive removal of denaturant and incubation at near-neutral pH, moderate ionic strength, and a protein concentration of 355 μM, the TIA1 LC domain forms both amorphous and fibrillar aggregates. Figure 1D shows that repetitive seeding in the absence of salt reduces the prevalence of the amorphous aggregates, producing solutions of TIA1 LC domain that contain long and somewhat bundled fibrils. These TIA1 LC domain fibrils appear similar to those from a previously published report,^18^ although differences in staining and image quality prevent any further comparison. The average fibril width in the TEM micrographs is 12.8 ± 2.4 nm. Additional fibril TEM images and an illustration of the fibril width measurement are shown in Figure S1. The residual soluble protein concentration for the seeded preparation is 1.4 μM. Using a model where fibrils do not fragment,^30^ this concentration corresponds to a Gibbs energy of dissociation (Δ*G*) for a TIA1 LC domain monomer from the fibril of ∼32 kJ/mol at ∼283 K.

### Residues A338–N357 of the TIA1 LC Domain Form β-Strand Rich Protein Fibrils

**Figure 2:**
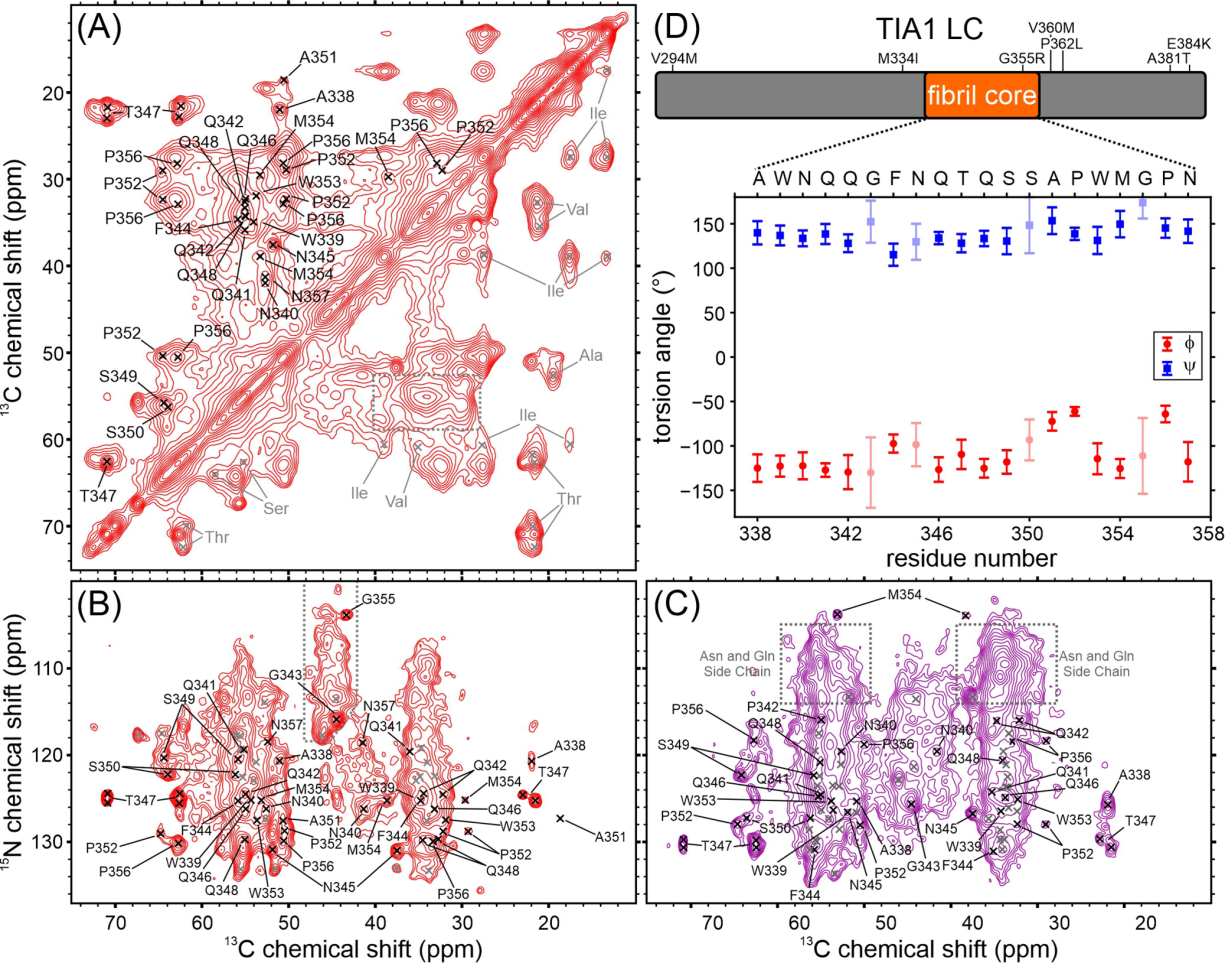
Solid state NMR characterization of seeded TIA1 LC domain fibrils. (A) Aliphatic region of a carbon–carbon cross-polarization-based correlation spectrum of seeded TIA1 LC domain fibrils. The spectrum is pseudosymmetric about the diagonal. Residues labeled in gray in the bottom right of the spectrum were not observed in nitrogen–carbon cross-polarization-based spectra. The gray dashed box highlights the overlapped signal intensity for Gln, Glu, Phe, Tyr, Trp, His, Met, Arg, and Leu residues. (B) Aliphatic region of a nitrogen–carbon cross-polarization-based spectrum showing intraresidue correlations. Residues marked in gray were not unambiguously assigned in this work. The gray dashed outline highlights three sharp signals and a region of broad signal intensity arising from Gly residues. (C) Aliphatic region of a nitrogen–carbon cross-polarization-based correlation spectrum showing interresidue correlations. Residues marked in gray were not unambiguously assigned in this work. The signal intensities indicated with gray dashed boxes originate from nitrogen atoms in Gln and Asn side chains. For all spectra, residues labeled in black were unambiguously assigned in this work. (D) Map of the TIA1 LC domain with the locations of the rigid fibril core and disease mutations and a plot showing the backbone torsion angles predicted from the assigned NMR chemical shifts. Error bars in the plot represent the standard deviation of the predictions, and lighter symbols indicate predictions with higher uncertainty.

Figure 2 shows the cross-polarization-based solid state NMR spectra of the hydrated seeded TIA1 LC domain fibrils, corresponding to those shown in Figure 1D. The spectra in Figure 2 exhibit a mixture of sharp, resolved signals and regions of either highly overlapped or broad signal intensity. Signals in these spectra arise from immobilized amino acids in the fibril structure. The 2D carbon–carbon correlation spectrum^31,32^ in Figure 2A contains sharp aliphatic signals for Thr, Ser, Ala, Asn, and Asp residues. The spectral regions corresponding to Gln, Glu, Phe, Tyr, Trp, His, Met, Arg, and Leu residues contain broad intensities which arise from either conformational heterogeneity of single residues, molecular motions, or the presence of multiple well-ordered residues. There are also broad resonances for all side-chain carbons of Pro residues. The spectrum also contains signal intensities consistent with Val and Ile residues. Notably, the signals corresponding to CA–CB correlations for the Val and Ile residues are significantly weaker than the signals corresponding to CB–CG and other terminal side-chain correlations, which suggest that the backbone atoms for the Val and Ile residues are not as well ordered as the side chains. The aliphatic-carbonyl region of this spectrum in Figure S2A shows at least two sharp Gly signals and a region of broad Gly signal intensity, although an analysis of this region is complicated by the presence of a spinning sideband. The aromatic–aliphatic region and aromatic–aromatic region of the spectrum in Figure S2A′–A″ show broad and weak signals and consistent backbone-sidechain correlations for Trp, Tyr, and Phe residues but lack any strong signals arising from His residues. The weak signal intensity in the aromatic region is similar to the other LC domain fibrils we have studied using solid state NMR.^26−28^ Weak aromatic side-chain signals are due to either poor internuclear magnetization transfers due to motionally averaged dipolar couplings or the aromatic side chains acting as a magnetization “sink” leading to a loss of magnetization in Phe and Tyr residues.^33^

The 2D cross-polarization-based nitrogen–carbon spectrum^22^ in Figure 2B more clearly reports on rigid Gly residues in the TIA1 LC domain. Sharp signals are consistent with at least two well-ordered Gly residues. Additional weak and broad signal intensity in this area is consistent with Gly residues that are either undergoing molecular motion or exist in a range of heterogeneous conformations. Much of the remainder of the spectrum is highly overlapped, but sharp signals can be identified for two Thr, three Ala, four Ser, and three Asn or Asp residues due to their unique NMR CA and CB chemical shift ranges. The narrow linewidths of these signals indicate they are rigid with well-defined molecular conformations. Pro residues lack an amide proton and therefore do not give rise to strong signals in the spectrum in Figure 2B. The TEDOR experiment^23^ ensures accurate reporting of all rigid Pro residues in nitrogen–carbon spectra, as it uses magnetization transfers originating from the CA proton rather than the amide proton. The TEDOR spectrum of the TIA1 LC domain fibrils in Figure S2B contains two sharp and distinct Pro signals with ^15^N chemical shift values of ∼130 ppm that arise from sites that are in well-defined and rigid conformations. These are consistent with weak Pro signal intensities in the cross-polarization-based spectrum in Figure 2B. There is additional broad signal intensity at larger ^15^N chemical shift values of ∼135–140 ppm consistent with Pro residues that are either loosely ordered or sample heterogeneous conformations. There is no signal intensity corresponding to these heterogeneous Pro residues in the cross-polarization-based spectrum in Figure 2B. The remainder of the TEDOR spectrum is otherwise consistent with the spectrum in Figure 2B.

The proton-carbon spectrum shown in Figure S2C uses scalar magnetization transfers^34^ that arise from highly mobile regions of the fibril structure. There is a signal in the spectrum with random coil NMR chemical shifts that can be unambiguously assigned to a Thr CB site. Signals from aromatic atoms in His sidechains are also present in the spectrum. The remainder of the signals in the spectrum arise from other aliphatic carbons that cannot be unambiguously assigned.

A comparison of the amino acid types observed in the spectra in Figure 2A,B, and Figure S2B with the protein sequence in Figure 1B reveals that the Ala, Gln, Pro, Trp, and Met signals arising from immobilized residues in the fibril structure are potentially spread along the entire TIA1 LC domain. However, the increased mobility of the backbone atoms for Ile and Val residues (Figure 2A) and their location in the N-terminal region of the TIA1 LC domain suggest that this region is not as uniformly ordered or immobilized as the C-terminal region, which is populated with Ala, Ser, and Thr residues that give rise to relatively sharp signals arising from well-ordered and rigid sites in the TIA1 LC domain. The small number of signals in the scalar-based spectrum in Figure S1C indicates that very few sites in the TIA1 LC domain are highly mobile. In our TIA1 LC construct, the only His residues and two additional Thr residues are present in the His-tag (see the Experimental Section, in the Supporting Information) used to express and isolate the TIA1 LC domain. The lack of strong signals from His residues in the cross polarization-based spectra indicate that these sites are not part of the rigid fibril core. Furthermore, the unambiguous signals from His sidechain sites and a Thr CB site in the spectrum in Figure S1B reinforce the interpretation that the tag does not influence the structure of the rigid fibril for the TIA1 LC domain. The 2D spectra presented in Figures 2A,B, S2B, and S2C are therefore consistent with a TIA1 LC domain fibril structure formed by a strongly immobilized and well-ordered core composed of but not limited to Ala, Thr, Ser, and Asn or Asp residues, with the remainder of the TIA1 LC domain exhibiting structural heterogeneity and limited motion, and the His-tag undergoing more rapid motion in a random coil-like configuration.

A total of 33 residues were resolved using 3D cross-polarization based spectra. The signal to noise and resolution in these spectra are shown using representative 2D planes in Figure S3. Almost all spectral regions exhibit well-resolved signal intensities in the 3D spectra, although there is some overlap in the region reporting on CB and CG sites from Gln, Glu, Phe, Tyr, Trp, His, Met, Arg, and Leu residues. However, there are enough features to identify signals from 15 distinct residues. Additionally, signals unambiguously attributable to two Ala, three Gly, two Pro, five Asn or Asp, two Thr, and four Ser residues are clearly identified in these spectra. Signals for Leu, Val, or Ile are not observed in the 3D spectra. Figure S4 shows representative planes from the Gly region of the 3D NCACX spectrum relative to their position in the 2D NCACX spectrum in Figure 2B, supporting the presence of both sharp and broad signals. These 3D spectra include an NCOCX experiment that provides the interresidue correlations required to associate the observed signals with specific residues in the TIA1 LC domain sequence. The spectrum from a 2D version of this experiment is shown in Figure 2C. All sharp and well-resolved signals identified in these 3D spectra are listed in Tables S2–S4. These tables do not include weak and broad signals that are not consistent with significant ordering of neighboring residues, such as the many weak NCACX Gly signals shown in Figures S2A and S4 that do not have matching crosspeaks in the NCOCX and CANCO spectra.

Unambiguous and statistically significant sequence-specific assignments for residues 338–357 were obtained using the MCASSIGN Monte-Carlo simulated annealing algorithm.^24^ The procedure used is similar to our work on other LC domain protein fibrils.^25−28^ A complete description of the assignment calculations is presented in the Experimental Section. Preliminary calculations were run to help exclude weak signals that did not have matching peaks across all 3D spectra. These weak signals are interpreted to arise from loosely ordered segments of the TIA1 LC domain that are neither immobilized enough for efficient cross-polarization or mobile enough for strong signals to arise in scalar-based spectra. The MCASSIGN algorithm was run twice on the remaining signals, with each run composed of 50 separate calculations performed with a unique seed for the random number generator, to produce statistically significant assignments. Signals that were assigned 50 out of 50 times from the first run were used as input for the second round of 50 MCASSIGN calculations. The procedure resulted in unique assignments for residues 338–357 (i.e., the same signals were assigned to the same residue in all 50 second-round MCASSIGN calculations). A strip plot showing the connectivity of the signal assignments resulting from the MCASSIGN algorithm is shown in Figure S5. The signals for F344 are relatively weak and the correlations for this residue are not observed in the CANCO spectrum, which is consistent with either motionally averaged dipolar coupling strengths or the Phe side chain acting as a magnetization “sink”.^33^ As expected due to the lack of an amide proton for Pro residues, the NCOCX signals preceding P352 and P356 are missing, as are the NCACX signals for these residues. The aromatic-aliphatic sidechain correlations consistent with our assignments for W339 and W353 are shown in Figure S2A. Signals representing an amino acid sequence of XGXX (consistent with residues W309–W312 or I319–Y322) could not be unambiguously assigned due to the low signal to noise of the flanking residues, suggesting an ordered region surrounded by more mobile segments of the protein. Figure 2D shows the TALOS-N^35^ torsion angle predictions from the assigned carbon and nitrogen chemical shifts. The large positive φ and large negative ψ values indicate the immobilized residues are in β-strand conformations. The spectra in Figures 2 and S2–S5 therefore are consistent with a β-strand rich fibril structure formed by a 20-residue core composed of residues A338–N357, flanked regions that are not highly mobile and lack well-defined structure.

### Both Wild-Type and P362L Mutant TIA1 LC Domain Liquid Droplets Age into Protein Fibrils

**Figure 3:**
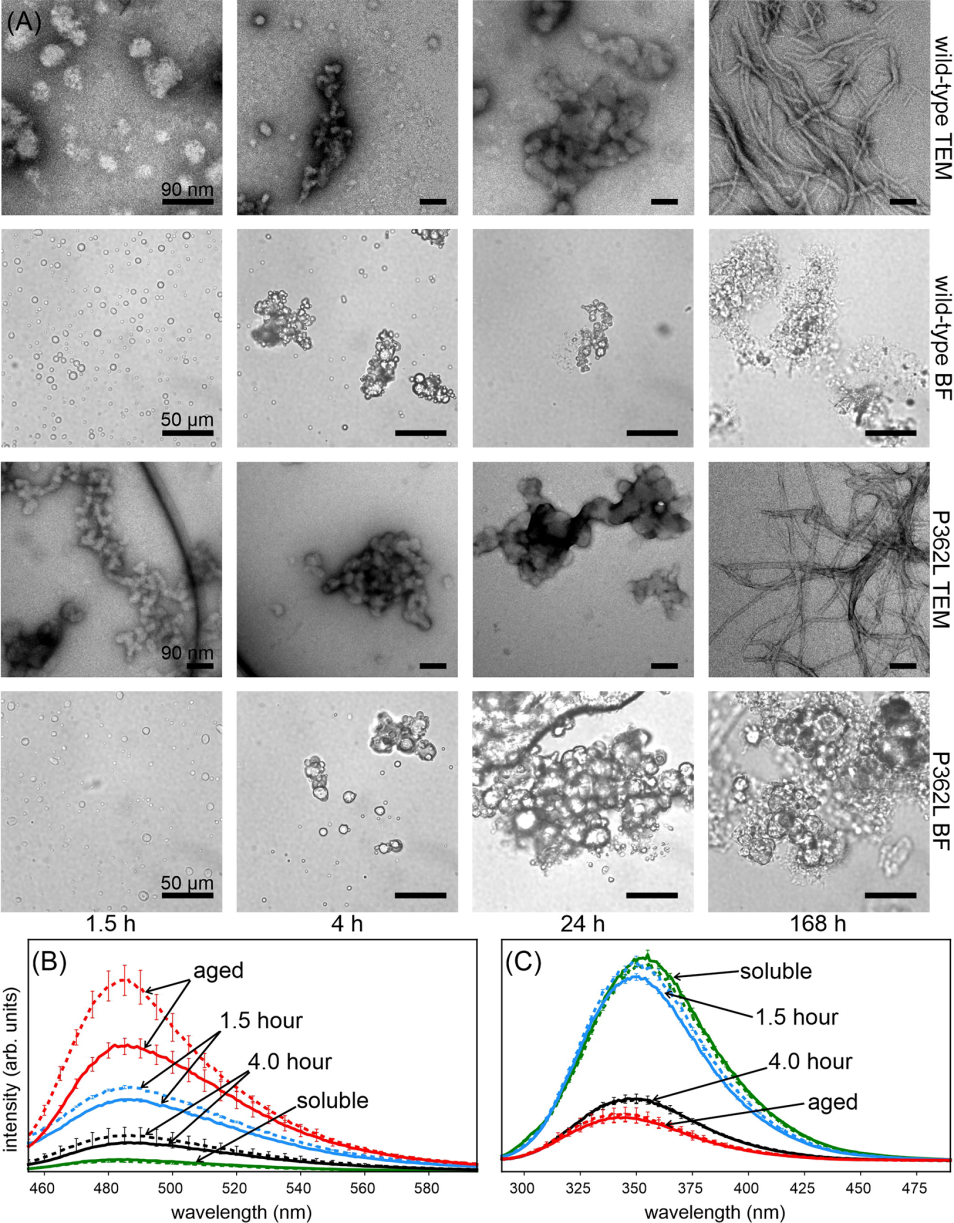
Liquid droplet aging of TIA1 LC domain wild-type and P362L mutant. (A) Bright field microscope images (BF) and negatively stained electron micrographs (TEM) of wild-type and P362L mutant TIA1 LC domain samples show the conversion of liquid droplets into amorphous aggregates that then convert into fibrils over the course of one week. (B) ThT fluorescence spectra recorded over the same time period show a slight increase in intensity after 1.5 h, which decreases at 4 h before increasing again over the course of a week. (C) Intrinsic Trp fluorescence spectra recorded over the same time period show a slight decrease in intensity and shift to lower wavelengths after 1.5 h, a trend that continues over the course of a week. In both (B) and (C), solid lines represent the data from wild-type TIA1 LC domain sample and dashed lines represent data from P362L mutant TIA1 LC domain sample. The error bars are the standard deviation of three measurements.

The bright field microscope images in Figure 3A show that removal of denaturant from purified solutions of wild-type and P362L mutant TIA1 LC domain at near neutral pH, moderate ionic strength, and a protein concentration of 83 μM produces solutions containing liquid droplets. The liquid droplets occur when the urea concentration drops below 1.5 M during dialysis^28^ and persist for up to 4 h. After 3 h the denaturant concentration is less than 200 mM. Movie S1 shows that at 1.5 h, when the denaturant concentration is ∼0.8 M, the liquid droplets fuse with one another, consistent with a condensed liquid droplet phase of mobile TIA1 LC domain protein. As the liquid droplets are allowed to age over 24 h, they clump together. Figure 3A also shows TEM images recorded during the aging process, which reveal the formation of amorphous aggregates concomitantly with the disappearance of the liquid droplets in the bright field images. After one week without agitation, TEM images primarily show long, thin fibrils with variable degrees of bundling. The average width of the fibrils in the wild-type aged liquid droplet sample is 13.4 ± 1.8 nm and the average width of the fibrils in the P362L mutant aged liquid droplet sample is 12.7 ± 1.4 nm. Additional TEM images of the aged liquid droplets are shown in Figure S1. The atomic force microscopy (AFM) images in Figure S6 also show that the large aggregates observed in the bright field images of wild type and P362L mutant aged liquid droplets contain large masses of highly bundled fibril-like structures. The residual soluble protein concentrations for the wild-type and P362L liquid droplet preparations were 1.4 μM, indicating the aged liquid droplets have similar thermodynamic stabilities as the seeded fibrils.

The ThT fluorescence spectra in Figure 3B show an increase in fluorescence intensity for both wild-type and P362L mutant TIA1 LC domain in condensed phases when compared to denaturant-solubilized protein. The intensity increases at 1.5 h, then decreases at 4 h before rising again at ∼1 week. An increase in ThT fluorescence suggests the presence of a β-strand rich conformation for TIA1 LC domain at 1.5 h and incubation times greater than 7 d, with reduced β-strand rich conformations at the intermediate 4 h time point. However, moderate increases in ThT fluorescence can arise from confinement of the fluorophore in a biomolecular condensate rather than the formation of rigid and extended β-strand structure.^28,36^ The confocal fluorescence microscopy images in Figure S7 show that at 1.5 h the ThT fluorescence signal arises from the liquid droplet species, while at 4 h, the images show that ThT fluorescence arises from hardened droplets and fibril-like protrusions extending from them. The confocal fluorescence microscopy images in Figure S6 show that the large masses of protein observed after 1 week for both wild type and P362L mutant samples all exhibit increased ThT fluorescence. Both the wild-type and P362L mutant TIA1 LC domain follow a similar ThT time course, suggesting the liquid droplet to fibril transition is similar for both samples. The total increase in the ThT intensity is larger for the P362L mutant sample than the wild-type sample and is consistent with previous ThT measurements on fibrils of full-length TIA1.^16^

Figure S8 shows that the magnitude of the ThT fluorescence signal from liquid droplet samples aged for at least 1 week is primarily dependent on the amount of bound ThT. Across multiple preparations of aged liquid droplets, we have observed variable absolute ThT intensities. Comparing the wild-type and P362L mutant aged liquid droplet samples we find: the fluorescence signal per bound ThT molecule is similar, our TEM and AFM images show predominantly fibril structures, our solid state NMR spectra show no difference in the molecular conformations sampled by the TIA1 LC domain (vide infra), and the residual soluble protein concentrations are similar. Therefore, the differences in ThT signal between the wild type and P362L mutant preparations are most likely due to variable degrees of fibril bundling and access of the ThT to the TIA1 LC domain molecules in a given sample rather than an increased amount of fibrils or differing fibril types.

The intrinsic tryptophan fluorescence spectra in Figure 3C show a steady decrease in intensity and a shift toward lower wavelengths over time for both wild-type and P362L mutant TIA1 LC domain. These spectra are consistent with one or more of the five Trp residues in the TIA1 LC domain having reduced accessibility to aqueous solvent.^37^ The large suppression of Trp fluorescence and distribution of the Trp residues in the TIA1 LC domain suggest that significant portions of the protein are protected from the solvent in the fibrils and to a lesser extent in the liquid droplets. The similar time-dependent Trp fluorescence signatures for both the wild-type and P362L mutant TIA1 LC domain indicate these processes are similar. The spectra at 4 h do not suggest an intermediate state with highly solvent exposed Trp residues as the liquid droplets age.

The ThT and Trp fluorescence measurements in Figure 3B,C are therefore consistent with both wild-type and P362L mutant TIA1 LC domains forming liquid droplets that transition into β-strand rich fibrils through an amorphous aggregate state on similar timescales.

### Wild-Type and P362L Mutant TIA1 LC Domain Aged Liquid Droplets Are Structurally Distinct from the Seeded Fibrils

**Figure 4:**
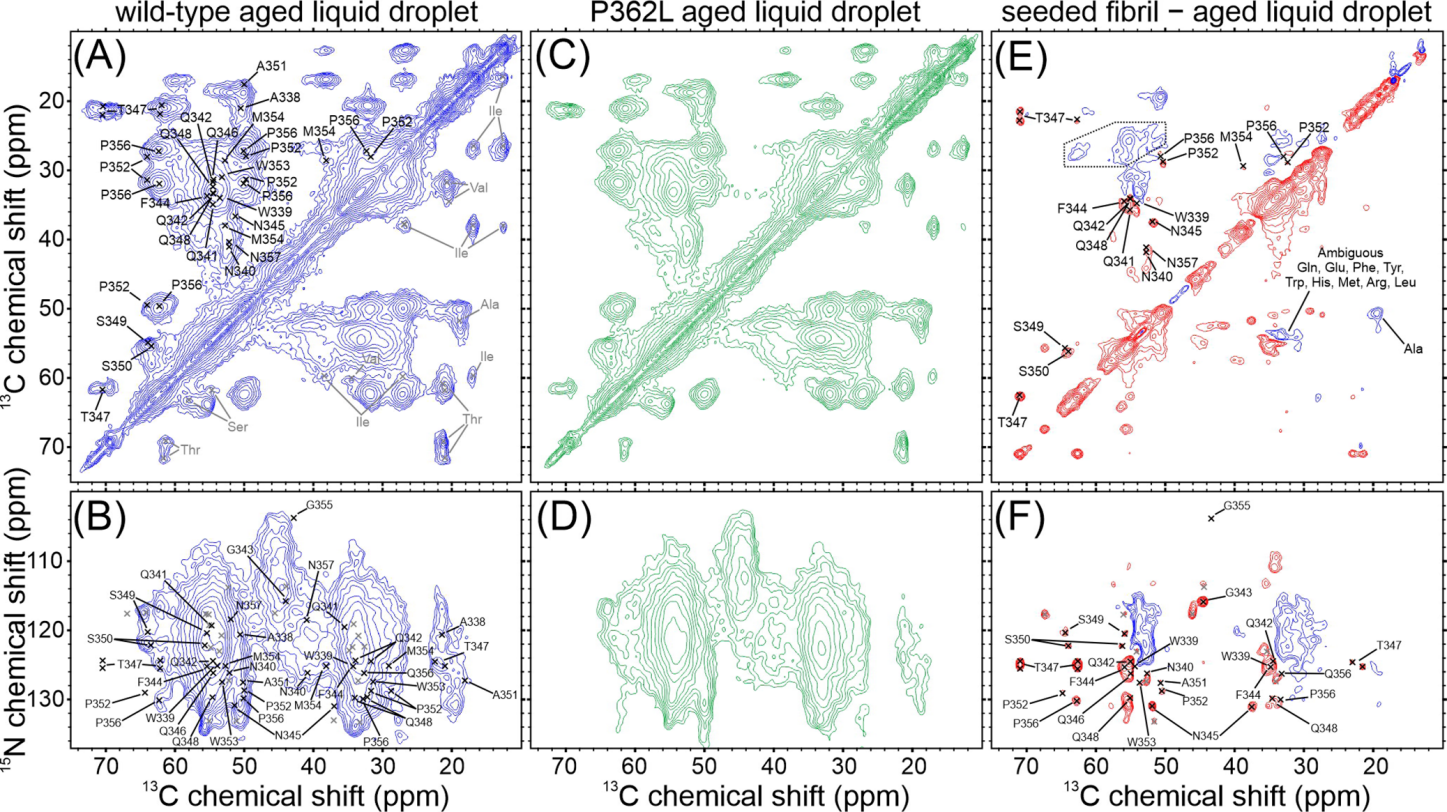
Solid state NMR characterization of wild-type and P362L mutant TIA1 LC domain aged liquid droplets. (A,C) Carbon–carbon cross-polarization-based correlation spectra of wild-type and P362L mutant TIA1 LC domain aged liquid droplets. (B,D) Nitrogen–carbon cross-polarization-based correlation spectra of wild-type and P362L mutant TIA1 LC domain aged liquid droplets. (E,F) Carbon–carbon and nitrogen–carbon cross-polarization-based difference spectra obtained by subtracting a spectrum of the wild-type aged liquid droplets from a spectrum of the wild-type seeded fibrils. In the difference spectra, blue contours represent signals that are stronger in the wild-type aged liquid droplet sample and red contours represent signals that are stronger in the wild-type seeded fibril sample. The dashed outline in (E) indicates an experimental artifact arising from a spinning sideband. For all spectra, the black labels and marks are the unambiguously assigned signals from the seeded TIA1 LC domain fibrils, and the gray marks are the unassigned signals from the seeded TIA1 LC domain fibrils.

Figure 4 shows cross-polarization based solid state NMR spectra of hydrated wild-type and P362L mutant TIA1 LC domain aged liquid droplets. The samples were harvested for NMR measurements after the brightfield, TEM, and fluorescence measurements shown in Figure 3 were recorded. The images in the rightmost column of Figure 3A therefore show the state of the samples that the NMR measurements were performed on. The carbon–carbon correlation spectra in Figure 4A,C show signal intensities that are consistent with ordered Thr, Ser, Pro, Ala, Asn or Asp, Gln or Glu, Phe, Tyr, Trp, Met, and Arg residues. A difference spectrum, where the spectrum of one sample is subtracted from that of another, highlights the most significant differences between the two spectra. Figure S9 shows overlay and difference spectra for the wild-type and P362L mutant aged liquid droplets, revealing minimal conformational differences in the TIA1 LC domains forming rigid structures in these samples. Since these experiments report on all rigid molecules in the samples, these spectra indicate that there is no significant difference in the amount of fibrils present in the wild-type and P362L mutant aged liquid droplets. Overall, the aged liquid droplet spectra are similar to those of the seeded wild-type TIA1 LC domain fibrils in Figure 2A. However, the difference spectrum comparing the seeded fibril and aged liquid droplet spectra for wild-type TIA1 LC domain in Figure 4E shows that the sharp signals for residues W339–Q342, F344–N345, T347–S350, P352, M354, and P356–N357 are not present in the aged liquid droplet spectra. These spectra therefore indicate that the well-ordered conformation obtained from fibril seeding is largely not present in the aged liquid droplet samples. Overlays of the spectra used to calculate the difference spectrum in Figure 4E are shown in Figure S9.

The nitrogen–carbon cross-polarization-based spectra in Figure 4B,D for the wild-type and P362L mutant TIA1 LC domain aged liquid droplets are also highly similar to one another. An overlay of these spectra and the corresponding difference spectra are provided in Figure S9. TEDOR-based spectra are also shown in Figure S9 to probe the presence of rigid Pro residues. These spectra highlight the conformational similarity of the wild-type and P362L mutant aged liquid droplets. However, like the carbon–carbon spectra, the nitrogen–carbon difference spectrum for the wild-type seeded fibril and aged liquid droplet spectra in Figure 4F reveals that the strong, sharp signals for residues W339, Q342–W353, and G355–P356 are not present in the spectrum of the aged liquid droplets, indicating that the seeded fibril core structure is largely not present in the aged liquid droplet samples. Overlays of the spectra used to construct Figure 4F are shown in Figure S9.

The spectra in Figure S10 show that lowering the sample temperature for the aged liquid droplet samples does not significantly change the appearance of the cross-polarization- and TEDOR-based spectra. The ^1^H spectra of these samples in Figure S10 show that water in the samples is not frozen at the −20 °C sample temperature, consistent with freezing point depression caused by the presence of salt and protein. Reducing the temperature probes the presence of loosely ordered but conformationally homogenous structure in these samples.^25^ As molecular motions decrease with temperature, cross-polarization and TEDOR based magnetization transfers should be more efficient due to stronger dipolar coupling interactions. Notably, the signals reporting on the seeded wild-type fibril conformation do not appear in these spectra. These low temperature data are therefore not consistent with the presence of a loosely ordered structure similar to the seeded wild-type conformation and further support that there are significant structural differences in molecular conformations between the seeded fibril and aged liquid droplet TIA1 LC domain samples. The low temperature spectra of seeded TIA1 LC domain fibrils shown in Figure S10 exhibits slightly broader lineshapes than the spectra recorded at 10 °C, but have otherwise similar appearances, indicating that there is no additional ordering of the seeded TIA1 LC domain fibrils at −20 °C.

Scalar-based experiments were performed on the wild-type and P362L mutant TIA1 LC domain aged liquid droplets. No signals were observed in the spectra recorded under the same conditions as the seeded TIA1 LC domain fibrils. Lack of signal intensity in these spectra indicate that there are no residues with significant molecular motion in either aged liquid droplet sample. The absence of signals from the His-tag in these spectra suggest that the His-tag has reduced mobility relative to the seeded sample. However, the absence of strong or sharp His signals in the cross-polarization-based spectra of the aged liquid droplets indicate that the His-tag has not adopted a rigid conformation either.

Cross-polarization-based ^15^N and ^13^C relaxation measurements for these samples probe the presence of structural heterogeneity and molecular motion in the samples. Table 1 shows the bulk ^15^N and ^13^C *T*_2_ values measured with 1D experiments for the wild-type and P362L mutant aged liquid droplets, and the seeded wild-type fibrils. Also shown in Table 1 and Figure S11 are ^15^N *T*_2_ measurements for resolved, sharp signals and regions of weak, broad signal intensity in a 2D NCACX spectrum. The bulk measurements indicate that the intrinsic ^15^N and ^13^C linewidths (full width at half maximum) for the aged liquid droplet and seeded samples are samples are on the order of 0.3–0.5 ppm. The measurements from the 2D NCACX spectra show that the sharp signals have even narrower ^15^N intrinsic linewidths, while the broad signals have similar values as those in the aged liquid droplet samples. In the context of the 2D solid state NMR spectra shown in Figure 4, these relaxation parameters are consistent with the TIA1 LC domain monomers in the aged liquid droplets occupying an array of heterogeneous rigid conformations rather than a single conformation undergoing molecular motion. In addition, the loosely ordered segments of the TIA1 LC domain that give rise to the broad unassigned signals in the cross-polarization-based spectra of the seeded fibrils have similar conformational properties as the protein in the aged liquid droplets.

**Table 1:** NMR Relaxation Measurements of TIA1 LC Domain Aged Liquid Droplets and Seeded Fibrils

site and sample	^13^C *T*_2_ (ms)	^15^N *T*_2_ (ms)
bulk, wild-type aged liquid droplet	2.92 ± 0.01	9.80 ± 0.02
bulk, P362L aged liquid droplet	2.93 ± 0.01	9.91 ± 0.02
bulk, wild-type seeded fibril	3.22 ± 0.02	14.1 ± 0.01
sharp signals, wild-type seeded fibril^a^		27.4 ± 1.8
broad signals, wild-type seeded fibril^a^		10.1 ± 1.1

a The average of the individual ^15^N *T*_2_ measurements.

## Discussion

Here we have shown, (i) a seeded preparation of the TIA1 LC domain yields uniform fibrils (Figures 1 and S1), (ii) the seeded fibril core is formed by residues A338–N357 in β-strand conformations (Figures 2 and S2–S5), (iii) aging of TIA1 LC domain liquid droplets results in β-strand rich fibrils that are conformationally heterogeneous and structurally distinct from the seeded fibrils (Figures 3, 4, S6, and S9), and (iv) the P362L mutation lies outside of the core forming region for the seeded fibrils and does not alter the structural characteristics of aged droplets (Figures 2, 4, and S9).

### Structure in Aged Liquid Droplets

Solid state NMR studies of condensate rigidification are currently limited in number but are capable of providing highly informative atomic-resolution or residue-specific characterizations of molecular conformation and motion. Heterochromatin protein 1α (HP1α) was shown to undergo a liquid droplet rigidification characterized by increased uniform and rigid structure coexisting with highly mobile and disordered structure.^38^ However, the detailed conformation of the HP1α protein in the aged liquid droplet sample was not characterized in the study. Aging of the fused in sarcoma (FUS) LC domain showed a similar increase of rigid structure in the presence of highly mobile and disordered regions of the protein.^39^ In this case, the aged liquid droplets were predominantly composed of a single rigid molecular conformation with NMR chemical shifts indicative of β-strand structures, results that were supported by TEM images and ThT positive fluorescence measurements.^39^ Although the molecular conformation of the FUS LC domain in the aged liquid droplets was similar to seeded FUS LC domain fibrils,^27^ significant differences in the solid state NMR spectra suggest the structural conversion was incomplete. Our recent study of aged liquid droplets of the TAR DNA-binding protein 43 (TDP43) LC domain revealed the protein converges on a singular β-strand rich molecular conformation without seeding.^28^ In these aged liquid droplets however, there was evidence for conformational heterogeneity and only a small fraction of the LC domain exhibited rapid molecular motion.^28^

Our measurements show the TIA1 LC domain aged liquid droplets exhibit strong ThT fluorescence relative to the newly formed liquid droplets, which is consistent with an increased amount of β-strand structure. However, the individual molecules in the aged liquid droplets are conformationally heterogeneous given the lack of strong and sharp peaks in the solid state NMR spectra, in contrast to the more conformationally homogenous aged liquid droplets of the FUS and TDP43 LC domains. Furthermore, significant portions of the TIA1 LC domain in both the seeded fibrils and aged liquid droplets do not participate in the rigid core of the structure but have limited molecular motion and are likely loosely ordered, characteristics that differ from the HP1α protein and FUS LC domain but are similar to the TDP43 LC domain. We also note the result that the ThT and Trp fluorescence spectra and TEM images of the TIA1 LC domain liquid droplet aging process suggest a non-fibrillar aggregate intermediate between the liquid droplet and β-strand rich fibril states, which was not observed for the HP1α, FUS-LC domain, or TDP43-LC domain proteins.^28,38,39^

Fibril seeding propagates specific fibril conformations for amyloid proteins like β-amyloid (Aβ), Tau, and α-synuclein.^40−42^ The fibril conformation that has the fastest propagation rate is selected, which may also be the most thermodynamically stable molecular conformation.^29^ However, different Aβ fibril polymorphs exhibit similar thermodynamic stabilities.^43^ While the TEM images and fluorescence data shown here indicate that the TIA1 LC domain liquid droplets age into predominantly fibril structures visually similar to the seeded TIA1 LC domain, our solid state NMR measurements show the aged liquid droplets exhibit a significant degree of conformational heterogeneity, contain a limited amount of the molecular conformation selected through seeding, and are inconsistent with the presence of another dominant conformation. These results suggest that the TIA1 LC domain is polymorphic regarding the formation of fibril structures, and our residual concentration measurements indicate that these conformations all have similar thermodynamic stabilities. Our observations suggest the intriguing possibility that the TIA1 LC domain conformations in aged liquid droplets might be structurally compatible and could coexist within the same fibril.

### Fibril Forming Cores of LC Protein Domains

Residues A338–N357 of the TIA1 LC domain have both differences and similarities with other fibril forming LC domain proteins. For the monomorphic LC domains, both the FUS^27^ and heterogeneous ribonucleoprotein A2 (hnRNPA2)^11^ LC domain fibrils are formed by longer 57-residue sequences. It has been proposed that monomorphic fibrils are functional.^48^ The amino acid content of these three LC domains is presented in Table 2. The Gly residues that can accommodate a larger range of backbone conformations are much more prevalent in FUS and hnRNPA2 than TIA1. There are more Asn and Gln residues able to form stabilizing polar zippers in TIA1 than FUS or hnRNPA2. A fibril core held together by Thr and Ser intramolecular hydrogen bonds^27,46^ is a defining feature for the FUS LC domain. While these residues do not seem to play the same dominant role in the hnRNPA2 fibrils, these residues are abundant in the TIA1 LC domain fibril core. Aromatic residues that interact through pi–pi interactions^44^ are similarly present in all three LC domain fibril cores. Regarding the hydrophobic aliphatic residues that stabilize the cores of many pathological amyloid fibrils, these residues are scarce in the FUS and hnRNPA2 fibrils. The TIA1 LC domain fibril core is enriched in these residues and is more like the TDP43 protein in this regard. The TDP43 LC domain is polymorphic,^28,49−51^ which is a feature of pathogenic amyloid fibrils.^47^ The higher prevalence of hydrophobic residues in the TIA1 LC domain fibril core compared to the FUS, hnRNPA2, and other similar LC domains may suggest that the propensity for liquid droplets to mature into polymorphic fibrils is likely to be more pathogenic than functional. However, the lack of TIA1 positive inclusions in patient tissues^16^ points to a more complicated role for TIA1 than simply forming pathogenic fibrils. One point worthy of further investigation is the lack of highly mobile regions outside the rigid core for the TIA1 LC domain fibrils. Longer rigid core regions, such as those in the FUS LC domain fibrils, sometimes have highly mobile flanking regions. It could be that the loosely ordered regions in the TIA1 LC domain fibrils provide extra stability for a shorter rigid core.

**Table 2:** Fractional Amino Acid Content of Fibril Core Forming Segments of LC Domains

residue type	TIA1 LC core^a^	FUS LC core^27^	hnRNPA2 LC core^11^
Gly	0.10	0.21	0.35
Asn/Gln	0.35	0.16	0.23
Ser/Thr	0.15	0.18	0.07
Pro	0.10	0.02	0.05
Aromatic	0.15	0.14	0.19
Aliphatic	0.25	0.02	0.07

a Values from this work.

### TIA1 LC Domain Disease Mutations, Liquid Droplets, and Fibrils

According to the results of our measurements, the P362L mutation in the TIA1 LC domain resides outside the seeded fibril core and does not appreciably alter the molecular conformations present in the aged liquid droplets. In the full-length TIA1 protein, the primary effect of the Pro-to-Leu mutations and other disease associated LC domain mutations are an increased persistence time of stress-induced granules and more rapid increases in ThT fluorescence.^16,18^ The overall persistence time of the wild-type and P362L mutant liquid droplets is shorter in our study of the TIA1 LC domain alone, but the rate of liquid droplet assembly is similar to the full-length TIA1 protein.^16^ The increased rate and magnitude of ThT fluorescence for the P362L mutant are also consistent with the full length TIA1 protein.^16^ Pro-to-hydrophobic mutations in the TIA1 LC domain have been proposed to promote extension of β-strand structures.^18^ While our study does not directly address the structure of seeded TIA1 LC domain P362L mutant fibrils, our results clearly show that the seeded wild-type TIA1 LC domain fibrils can accommodate uniform and rigid conformations for Pro residues (P352 and P356). Although Pro residues typically terminate β-strand structures, as observed in the FUS LC domain fibril structure,^27^ Pro residues can be accommodated in β-strand structures as observed in a fragment of Transthyretin (TTR).^52^ The observed chemical shifts for the TTR, and FUS and hnRNPA2 LC domain fibrils are compared to those observed for the TIA1 LC domain in Table 3. The published structures show the FUS LC and TTR Pro residues are in the trans conformation. The similarity of the chemical shifts for all LC domains in Table 3 suggest that for the LC domain fibrils studied to date with rigid Pro residues, the Pro are in the trans conformation. Furthermore, ^13^CG chemical shifts are particularly sensitive to these conformations with values between ∼24–25 ppm indicative of cis conformations.^57^ Based on this metric, none of the Pro residues listed in Table 3 are in a cis conformation. Any potential role of cis–trans isomerization in LC domain fibrils remains unaddressed.

**Table 3:** Proline NMR Chemical Shifts Observed in Fibrils

residue	^15^N (ppm)	^13^CO (ppm)	^13^CA (ppm)	^13^CB (ppm)	^13^CG (ppm)	^13^CD (ppm)	conformation
TIA1 LC (P352)^a^	129.0	174.3	64.7	32.5	29.1	50.3	unknown
TIA1 LC (P356)^a^	130.1	173.4	62.7	32.9	28.6	50.5	unknown
TTR (P113)^58^	135.8	174.8	62.6	32.6	28.0	49.6	trans
FUS LC (P72)^27^	132.9	176.6	62.3	32.3	28.5	47.2	trans
hnRNPA2 LC (P298)^26^	133.3	176.9	63.0	32.4	28.8	49.2	unknown
hnRNPA2 LC (P303)^26^	134.7	176.8	63.3	32.3	28.9	49.4	unknown
hnRNPA2 LC (P318)^26^	137.7	178.5	63.8	32.0	29.6	51.1	unknown

a Values from this work.

**Figure 5:**
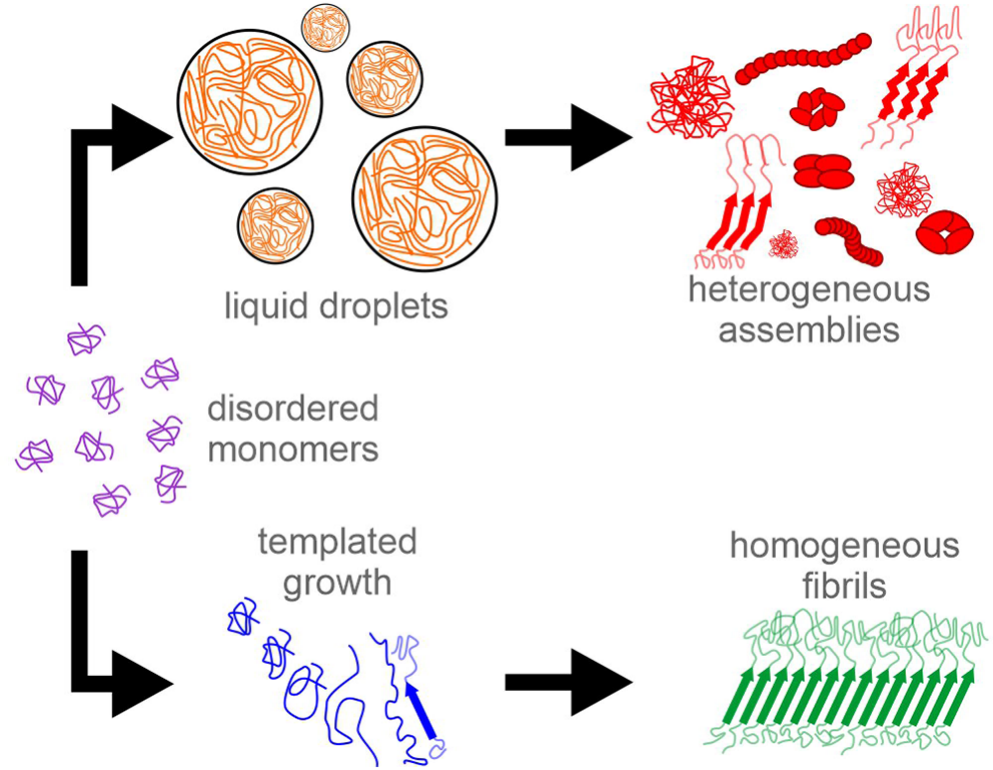
Potential assembly pathways for the TIA1 LC domain. In one pathway, monomeric protein can condense into liquid droplets in response to stress stimuli. The macroscopic rigidification of the liquid droplets is due to the protein molecules adopting heterogenous conformations that may include protofibrils, oligomers, fibrils, and amorphous aggregates. In a second pathway, seeds composed of molecules in well-defined conformations can template limited homogeneous fibril formation to bring proteins together functionally.

Furthermore, the heterogeneous molecular conformations we observe for the aged wild-type and P362L mutant liquid droplets are not appreciably different. Given the similarities in maturation kinetics for our TIA1 LC domain liquid droplets with the full-length TIA1 protein, we propose our data support that the P362L mutation mainly affects LC domain protein–protein interactions in the dynamic liquid droplet state rather thermodynamically favoring a specific rigid fibrillar conformation. The increased persistence time and altered molecular interactions inside the liquid droplets caused by mutations may therefore be responsible for nucleating aggregation of other proteins like TDP43. However, our study of the isolated LC domain does not unambiguously rule out that the P362L mutation in the full-length protein could also disrupt interactions between the LC domain and the N-terminal RNA-binding domains. We also note that considerable discussion has been devoted to the functional role of limited seeded TIA1 fibril formation.^20^ Our detailed characterization of the seeded fibril core may therefore provide a starting point for a more precise dissection of functional TIA1 assembly. Figure 5 depicts the two separate pathways for TIA1 LC domain assembly that are suggested by our results.

Finally, it is important to note that the full-length TIA1 protein has been observed *in vitro* to form micellar structures mediated by interactions with the N-terminal RNA-binding domains,^53^ rather than the LC domain studied here. While it is probable that all potential interactions for the TIA1 protein are relevant in living cells, the LC domain is a key component of *in vivo* behavior, as observed in altered stress granule dynamics due to LC domain mutations.^16^ Determining the balance of N-terminal oligomerization and C-terminal fibril formation is a key next step in characterizing TIA1 function and pathology.

## Conclusions

Characterizing TIA1 LC domain assembly is an important component of a broad search for understanding other functional and pathological activities governed by LC domain protein self-assembly. 30% of proteins in the human proteome have at least one LC domain.^8^ The amino acid biases of these proteins vary greatly. Guiding principles are emerging for the role of amino acid types and distributions for liquid droplet formation.^54,55^ However, functional amyloid-like fibril formation is also a key component of organizing cellular activity.^56^ The large number of missense mutations in LC domains linked to disease^15^ make it clear that the precise sequences of these proteins are important. Since functional LC domains differ in amino acid content from those of traditional and purely pathological amyloids, further characterizations of these fascinating and ubiquitous protein domains are needed.

## Supporting Information Available

Video of liquid droplets (AVI)
Protein purification and plasmid mutagenesis methods; microscopy methods; fluorescence spectroscopy methods; additional TEM images; additional 2D solid state NMR spectra of seeded TIA1 LC domain fibrils; 2D planes from the 3D solid state NMR spectra of seeded TIA1 LC domain fibrils; solid state NMR spectra of sharp and broad Gly signals; strip plot of the assigned signals for the seeded TIA1 LC domain fibrils; AFM and confocal fluorescence fluorescence microscopy images; comparison of the seeded fibril and aged liquid droplet wild-type and P362L TIA1 LC domain spectra; low temperature spectra of the TIA1 LC domain samples; relaxation measurements from 2D spectra of seeded TIA1 LC domain fibrils; tables of MCASSIGN input tables; table of experimental conditions for solid state NMR data collection (PDF)

## Supplementary Material

ja2c08596_si_001.avi
