# Supplementary material for: Liquid Droplet Aging and Seeded Fibril Formation of the Cytotoxic Granule Associated RNA Binding Protein TIA1 Low Complexity Domain
Source: J Am Chem Soc. 2023 Jan 13;145(3):1580–92. doi: 10.1021/jacs.2c08596 (PMC9881004; doi:10.1021/jacs.2c08596)
Supplement: Supplementary file 2 — ja2c08596_si_002.pdf [file ja2c08596_si_002.pdf]

Liquid droplet aging and seeded fibril formation of the TIA1 stress granule protein low complexity domain

Yuuki Wittmer[1], Khaled M. Jami[1], Rachelle K. Stowell[1], Truc Le[1], Ivan Hung[2], Dylan T. Murray[1,*]

[1]Department of Chemistry, University of California, Davis, California 95616, USA
[2]National High Magnetic Field Laboratory, Tallahassee, Florida 32310, USA

Contents:

- Protein purification and plasmid mutagenesis methods
- details of microscopy measurements
- details of fluorescence spectroscopy
- video of liquid droplets
- additional TEM images
- additional 2D solid state NMR spectra of seeded TIA1 LC domain fibrils
- 2D planes from the 3D solid state NMR spectra of seeded TIA1 LC domain fibrils
- solid state NMR spectra of sharp and broad Gly signals
- strip plot of the assigned signals for the seeded TIA1 LC domain fibrils
- AFM and confocal fluorescence microscopy images of aged liquid droplet samples
- Confocal fluorescence microscopy images of liquid droplets at short time periods
- comparison of the seeded fibril and aged liquid droplet wild type and P362L TIA1 LC domain solid state NMR spectra
- low temperature spectra of the TIA1 LC domain samples
- relaxation measurements from 2D spectra of seeded TIA1 LC domain fibrils
- tables of MCASSIGN input tables
- table of experimental conditions for solid state NMR data collection.

Supplemental Experimental Section:

Protein Expression and Purification: His-tagged wild-type and P362L mutant TIA1 LC domains (residues I291–Q386) were recombinantly produced using a pHIS-parallel plasmid[21] that includes an N-terminal His-tag (MSYYHHHHHHDYDIPTTENLYFQGAMDPEF). The plasmid was transformed into BL21(DE3) *E. coli* cells. All cells were grown in a shaker incubator at 37 °C and 220 revolutions per minute (RPM). For unlabeled protein, bacterial cultures were grown in Luria broth media in the presence of 100 μg/ml ampicillin to an optical density at 600 nm of 0.6–1.0 measured using a 1 cm pathlength cuvette before adding isopropyl β-D-thiogalactopyranoside (IPTG) to 0.5 mM to induce protein expression. The cultures were grown for another 3 h before harvesting the cells by centrifugation at 6,000 g for 15 min. The cell pellets were flash frozen in liquid nitrogen and stored at −80 °C until purification. For ^{13}C and ^{15}N labeled protein, bacterial cells were grown to an optical density at 600 nm of ~2.0 in Dynamite Broth[22] with 100 μg/mL ampicillin. Cells from 2 L culture were harvested by centrifugation at 6,000 g for 10 min and transferred into 1 L of M9 minimal media using a serological pipette (45.8 mM Sodium phosphate dibasic heptahydrate, 22.0 mM Potassium phosphate monobasic, 8.6

mM sodium chloride, 2.0 mM magnesium chloride, 0.1 mM calcium chloride, and 100 μg/ml ampicillin 2.0 g of U-$^{13}C_6$ D-glucose, and 1.0 g of ^{15}N ammonium chloride (Cambridge Isotope Labs). Protein expression was induced after growing the cells for 0.5 h longer at 37 °C with 220 RPM shaking by adding IPTG to 0.5 mM. The cells were harvested 3 h later by centrifugation at 6,000 g for 15 min, flash frozen in liquid nitrogen, and stored at −80 °C until purification.

Purification procedures for the wild type and P362L mutant TIA1 LC domain were the same. Cell pellets were thawed on ice for ~15 min and resuspended in 6 M guanidinium hydrochloride, 50 mM tris(hydroxymethyl)aminomethane (Tris) pH 7.5, 500 mM sodium chloride, and 1% v/v Triton X-100 along with 3 pellets of Mini EDTA-free Pierce Protease Inhibitor (ThermoFisher Scientific), and 0.25 mg/ml hen egg white lysozyme. The resuspended cells were sonicated using a Branson 250 Sonifier equipped with a 1/4 inch microtip in an ice-water bath for a total of 20 min at 30% output, in cycles of 0.3 s on and 3 s off, for a total of 1 min sonication. The lysed cells were centrifuged at 4 °C and 75,600 g for 30 min to remove insoluble material. TIA1 LC domain was isolated from the supernatant using a Bio-Rad NGC Discover 10 chromatography system and a 5 mL Ni-affinity column (Bio-Rad Bio-scale Mini Nuvia IMAC or GE Healthcare HisTrap FF crude). The column was equilibrated in 500 mM sodium chloride, 6 M urea, and 20 mM 4-(2-hydroxyethyl)-1-piperazineethanesulfonic acid (HEPES) pH 7.5; washed in 500 mM sodium chloride, 6 M urea, 20 mM imidazole, and 20 mM HEPES, pH 7.5; and eluted in 500 mM sodium chloride, 6 M urea, and 20 mM HEPES, pH 7.5, using a 20–200 mM imidazole gradient. 1 mL aliquots of the elution peak containing TIA1 LC domain were prepared and stored at −80 °C. The presence and purity of TIA1 LC domain was confirmed by SDS-PAGE with Coomassie staining.

TIA1 LC Domain P362L Site-directed Mutagenesis: The P362L mutant His-tagged TIA1 LC domain plasmid was prepared using forward and reverse primers containing the P362L mutation (5′-ccattttgcccttgaggcagttgcactccataatttg-3′ and 5′-caaattatggagtgcaactgcctcaagggcaaaatgg-3′, Integrated DNA Technologies). A polymerase chain reaction (PCR) was performed by mixing 0.5 μL of the pHis-parallel wild-type TIA1 LC domain plasmid (123.1 ng/μl), 2.5 μL of 10 μM forward and reverse primers, 5× Phusion GC buffer, 1 μL of 10 mM deoxynucleotides, 1.5 μL of dimethyl sulfoxide, and 0.5 μL of a Phusion DNA polymerase was mixed gently in a 50 μl volume. Polymerase, buffer and dNTPs were purchased from New England BioLabs. PCR was performed using a Bio-Rad T100 thermal cycler with an initial denaturation step at 98 °C for 30 s followed by 30 cycles of 7 s denaturation at 98 °C, 20 s of annealing at 72 °C, and extension for 2 min 42 s, and finally a final extension for 8 min at 72 °C. DpnI (New England Biolabs) was added to the PCR reaction vial and incubated at 37 °C for 1 h before storage at 4 °C. The PCR product was transformed into DH5α *E. coli* cells and grown in Luria broth media for 17 h at 37 °C with 220 RPM shaking. The amplified plasmid was purified using a Qiagen QIAprep Spin Miniprep kit. The sequence of the plasmid was confirmed by Sanger sequencing (Genewiz).

Brightfield Microscopy: Liquid droplets were imaged on Olympus BX51 light microscope with differential interference contrast (DIC) filters, a 40× objective lens, and a Diagnostics Instruments RT Slider camera with 6-megapixel sampling. 3 μL of sample was dispensed onto a glass microscope slide and imaged immediately.

Transmission Electron Microscopy: Cu or Au 300–400 mesh lacey carbon grids with ultrathin carbon films (Ted Pella) were glow discharged before applying 5 μL of the TIA1 LC domain solutions and incubating for 2 min. Bulk solution was blotted using a tissue before washing the grid twice by applying 5 μL of ultrapure water for ~10 s and blotting away the water between each application. The grid was stained using 5 μL of 3% (w/v) uranyl acetate, incubated for ~10 s, blotted with a tissue, and air-dried. Images were obtained on a JEOL-1230 electron microscope equipped with a 2K × 2K Tietz CCD camera operating at 100 keV.

Atomic Force Microscopy: An aliquot of the aged liquid droplet sample was diluted to 17 μM with 20 mM HEPES, 150 mM sodium chloride, pH 7.5, before pipetting 5 μL of the dilution onto a freshly cleaved mica surface and incubating for 15 min. The bulk solution was then wicked away with a laboratory tissue and 300 μL of ultrapure water was added to wash the surface. Finally, the bulk water was wicked away again with a laboratory tissue and the surface was dried under a stream of dry nitrogen gas and then placed in a clean laminar flow hood for 30 min. The surface was imaged with an Oxford Instruments Asylum Research MFP3D Atomic Force Microscope. Samples were imaged in air under ambient conditions using an AC240 cantilever (Olympus) with a resonance frequency of 70 kHz and spring constant of 2 N/m. The drive amplitude was 1.0 V and the set point was 0.6 V. Height and phase images were recorded with 512 x 512 pixels.

Fluorescence Microscopy: For each measurement, 10 μL of the neat liquid droplet sample was mixed with 10 uL of a 40 μM ThT solution in 20 mM HEPES, 150 mM sodium chloride, pH 7.5 to give a solution of 42 μM TIA1 LC domain, 20 μM ThT, 20 mM HEPES, and 150 mM sodium chloride, pH 7.5. Both the wild-type and P362L mutant liquid droplets were imaged by placing 20 μL of the mixture onto a glass bottom MatTek dish (MatTek USA). Bright field and fluorescence images were recorded on an Olympus IX81 inverted microscope equipped with a FV1000 Laser Scanning Confocal Microscope system. Images were taken with a 60x oil immersion objective (Olympus PlanApo N 60x/1.42 na Oil Objective). Fluorescent confocal imaging used an Argon laser with an excitation wavelength of 458 nm (excitation dichroic mirror set to 458 nm/515 nm). Fluorescence emission was detected between 480 and 580 nm. Bright field images were recorded on a second channel parallel to the one used to record the fluorescence images. Z-stack time-lapse images were collected with a scan speed of 2.0 μs/pixel, a total area of 800 x 800 pixels, and a 0.5 μm slice thickness.

ThT and Intrinsic Fluorescence Assays: A saturated ThT stock solution (>200 μM, Acros Organics) was prepared in ultrapure water and sterile filtered (0.22 μM PES membrane, Millipore). The ThT working concentration of 40 μM with 150 mM sodium chloride and 20 mM HEPES, pH 7.5 was determined using absorbance at 412 nm (ε_{412}=36,000 $M^{-1}cm^{-1}$). All ThT solutions were stored at 4 °C, wrapped in foil, and used within 7 d. For ThT fluorescence measurements, 50 μL of a 40 μM ThT working solution was added directly into 50 μL of the protein sample. Intrinsic fluorescence measurements were performed on neat protein solutions. All measurements were recorded on an Agilent Cary Eclipse fluorescence spectrophotometer using a 45 μL 10 mm × 10

mm quartz cuvette (Starna) with 50 µL samples. The excitation and emission slit widths were 5 nm with a scan rate of 600 nm/min, an averaging time of 0.1 s, and an interval of 1.0 nm. Intrinsic fluorescence data were recorded with an excitation wavelength of 280 nm. ThT data were acquired with an excitation wavelength at 440 nm.

Supplemental Video 1: TIA1 LC Domain Wild-Type Liquid Droplet Dynamics. Wild-type TIA1 LC liquid droplets after 1.5 h of dialysis. Each frame is taken 1 s apart and the video covers 50 s of real time.

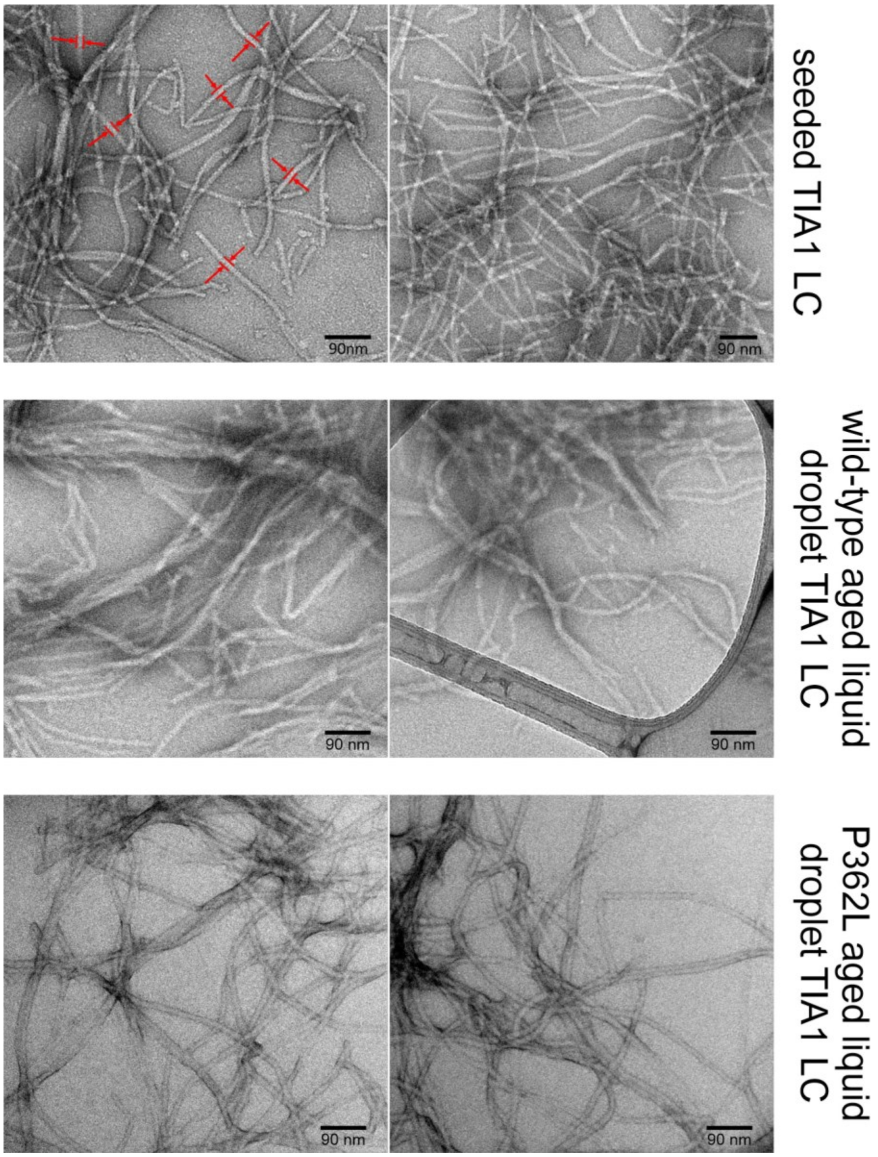

Supplemental Figure S1. Additional negatively stained TEM images of the TIA1 LC domain samples. The arrows in the top left image illustrate how the fibril width was measured.

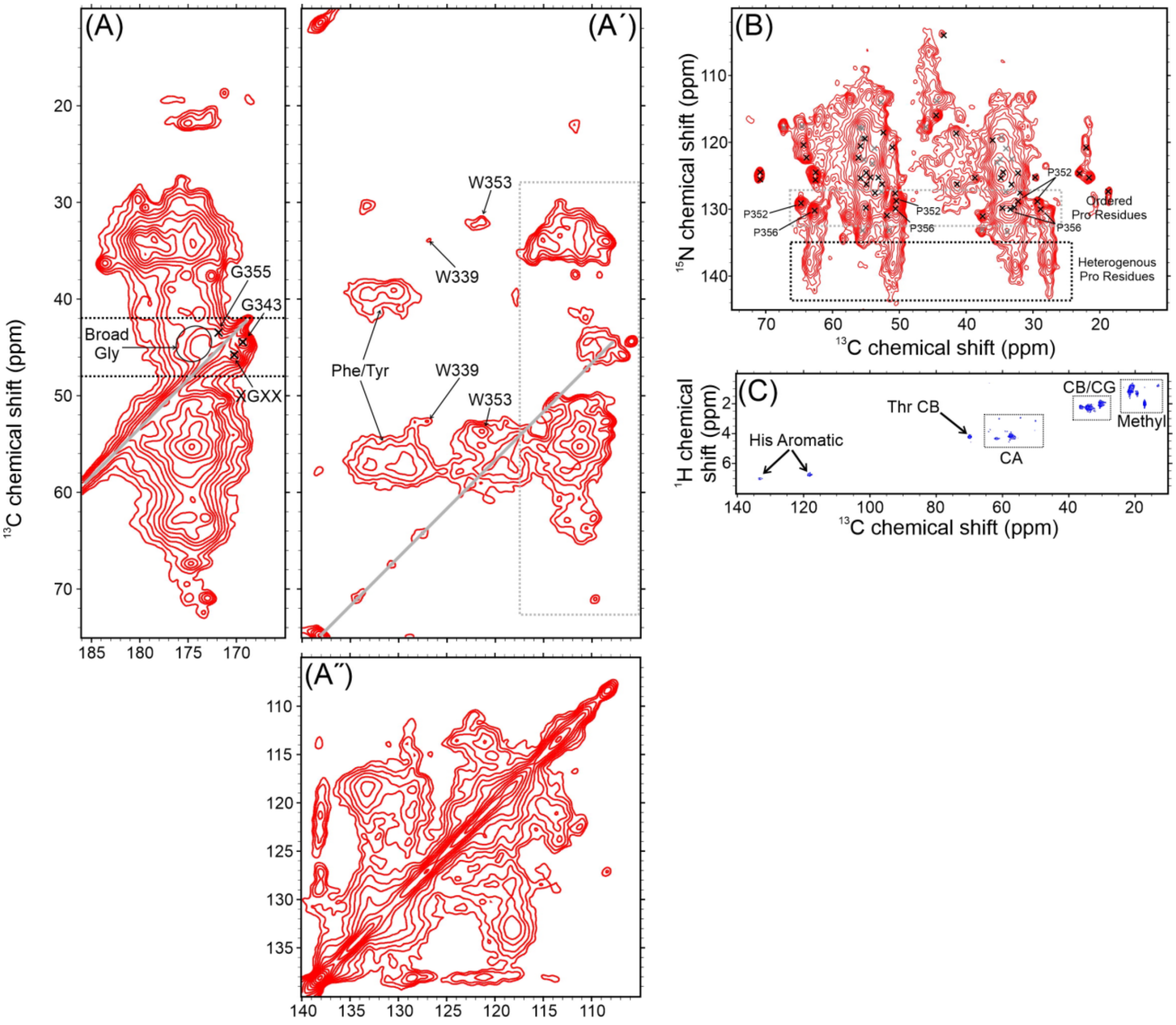

Supplemental Figure S2. Additional 2D Solid State NMR Spectra of the Seeded TIA1 LC Domain Fibrils. (A–A″) aliphatic-carbonyl, aliphatic-aromatic, and aromatic-aromatic regions of the cross polarization-based CC-DARR spectrum of wild-type seeded TIA1 LC fibrils. In (A) the three sharp Gly signals are labeled in addition to the weaker broad signal intensity from other Gly residues. The black dashed lines highlight the chemical shift range for the Gly signals observed in NCACX spectra of the seeded TIA1 LC domain fibrils. In (A′) broad intensities for unassigned Phe and Tyr sidechains are labeled, and the sidechain signals for the assigned Trp signals are labeled. In (A and A′) the gray line marks a spinning sideband and in (A′) the gray box marks further signals arising from spinning sidebands. (B) cross polarization-based 2D NCACX-TEDOR spectrum of the wild-type seeded TIA1 LC fibrils highlights ordered Pro residues. Pro residues with rigid homogeneous conformations are labeled and indicated with a gray dashed box. Pro residues with rigid but heterogeneous conformations are indicated with a black dashed box. Black marks are the unambiguously assigned signals and gray marks represent signals lacking unambiguous assignments for the wild type seeded TIA1 LC domain fibrils. (C) Scalar-based 2D ^{1}H-^{13}C INEPT spectrum of the wild-type seeded TIA1 LC fibrils

highlights highly mobile sites. Signals that can be unambiguously assigned to an amino acid type are two aromatic His sidechain sites and a Thr CB site. Other signals are observed from CA, CB or CG, and methyl groups in amino acid sidechains. The contours are drawn at intensity values increasing by a factor of 1.4 in (A–A”), 1.25 in (B), and 1.5 in (C).

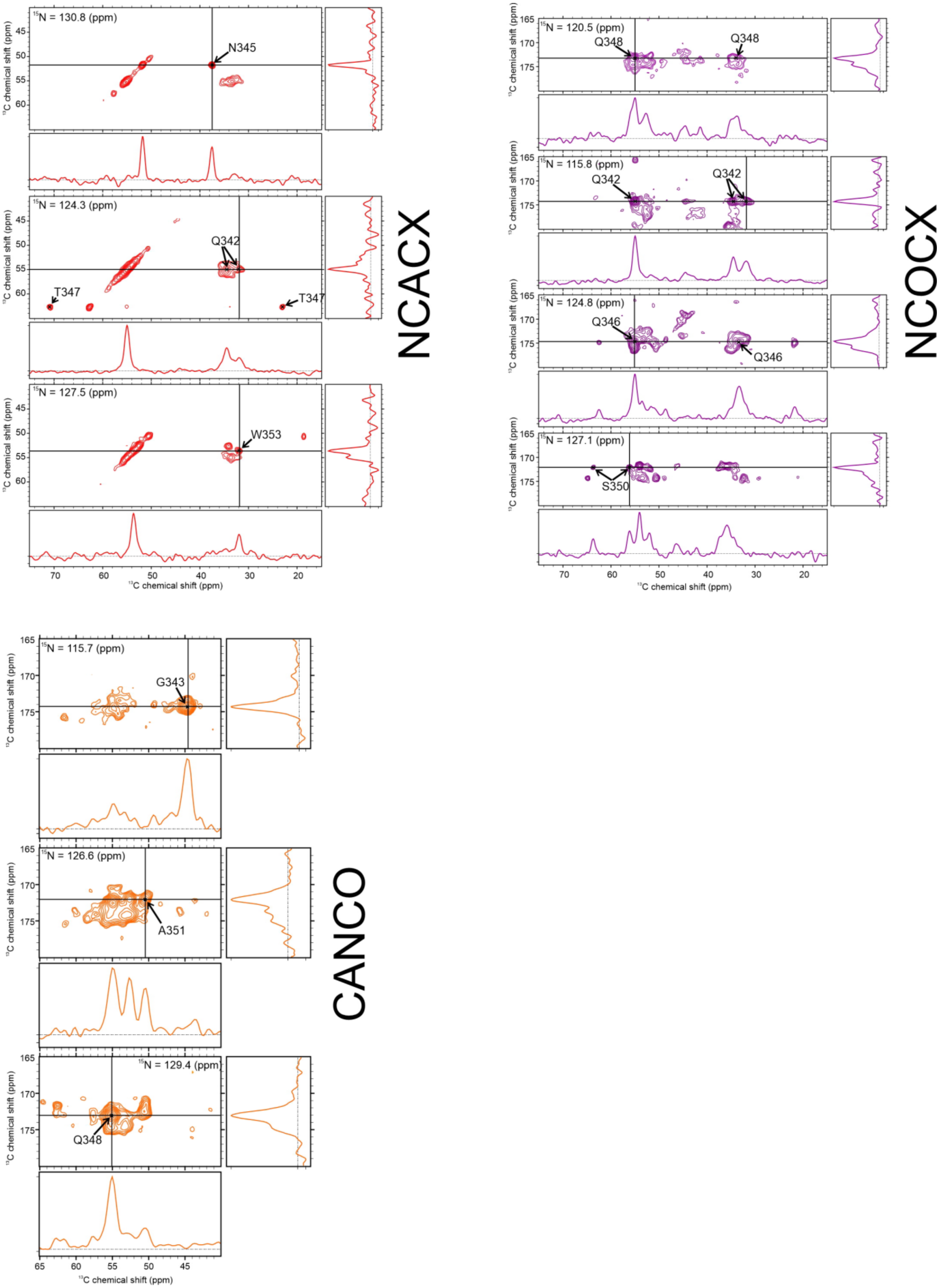
NCACX
15N = 130.8 (ppm)
N345
15N = 124.3 (ppm)
Q342
T347
T347
15N = 127.5 (ppm)
W353
13C chemical shift (ppm)
NCOCX
15N = 120.5 (ppm)
Q348
Q348
15N = 115.8 (ppm)
Q342
Q342
15N = 124.8 (ppm)
Q346
Q346
15N = 127.1 (ppm)
S350
13C chemical shift (ppm)
CANCO
15N = 115.7 (ppm)
G343
15N = 126.6 (ppm)
A351
15N = 129.4 (ppm)
Q348
13C chemical shift (ppm)

Supplemental Figure S3. 2D Planes From the 3D Spectra of the Wild-type Seeded TIA1 LC Domain Fibrils. These 2D planes demonstrate the signal to noise and increased resolution in the 3D cross polarization-based NCACX, NCOCX, and CANCO experiments. The side and bottom panels next to each plane contain slices from the data at the location of the horizontal and vertical lines. The contours are drawn at intensity values increasing by a factor of 1.3.

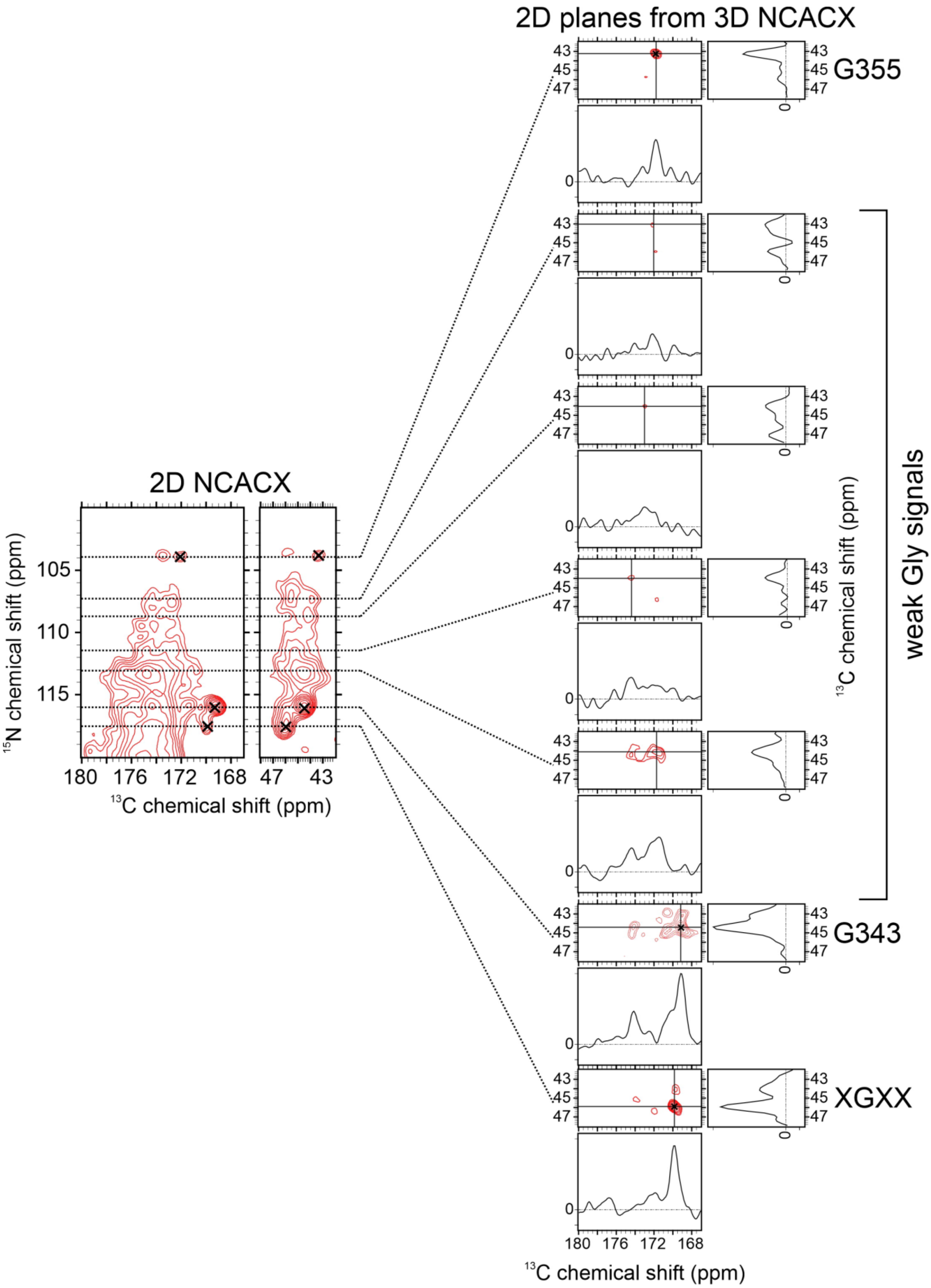

2D planes from 3D NCACX
2D NCACX
G355
G343
XGXX
weak Gly signals
^{15}N chemical shift (ppm)
^{13}C chemical shift (ppm)
^{13}C chemical shift (ppm)
^{13}C chemical shift (ppm)

Supplemental Figure S4. 2D Planes from the 3D NCACX Spectrum of Seeded TIA1 LC Domain Fibrils Highlight the Sharp and Broad Signals from Gly Residues. The Gly carbonyl and aliphatic regions of the 2D NCACX spectrum of seeded TIA1 LC domain fibrils are shown on the left. The horizontal lines highlight the ^{15}N frequencies at which the 2D planes on the right side of the figure are extracted from the 3D NCACX spectrum. The horizontal and vertical lines within these 2D planes illustrate the relative signal to noise of the sharp Gly resonances that are included in the MCASSIGN assignment calculations compared to the broad Gly resonances that are not included in the MCASSIGN assignment calculations. All 1D slices are plotted on the same vertical scale.

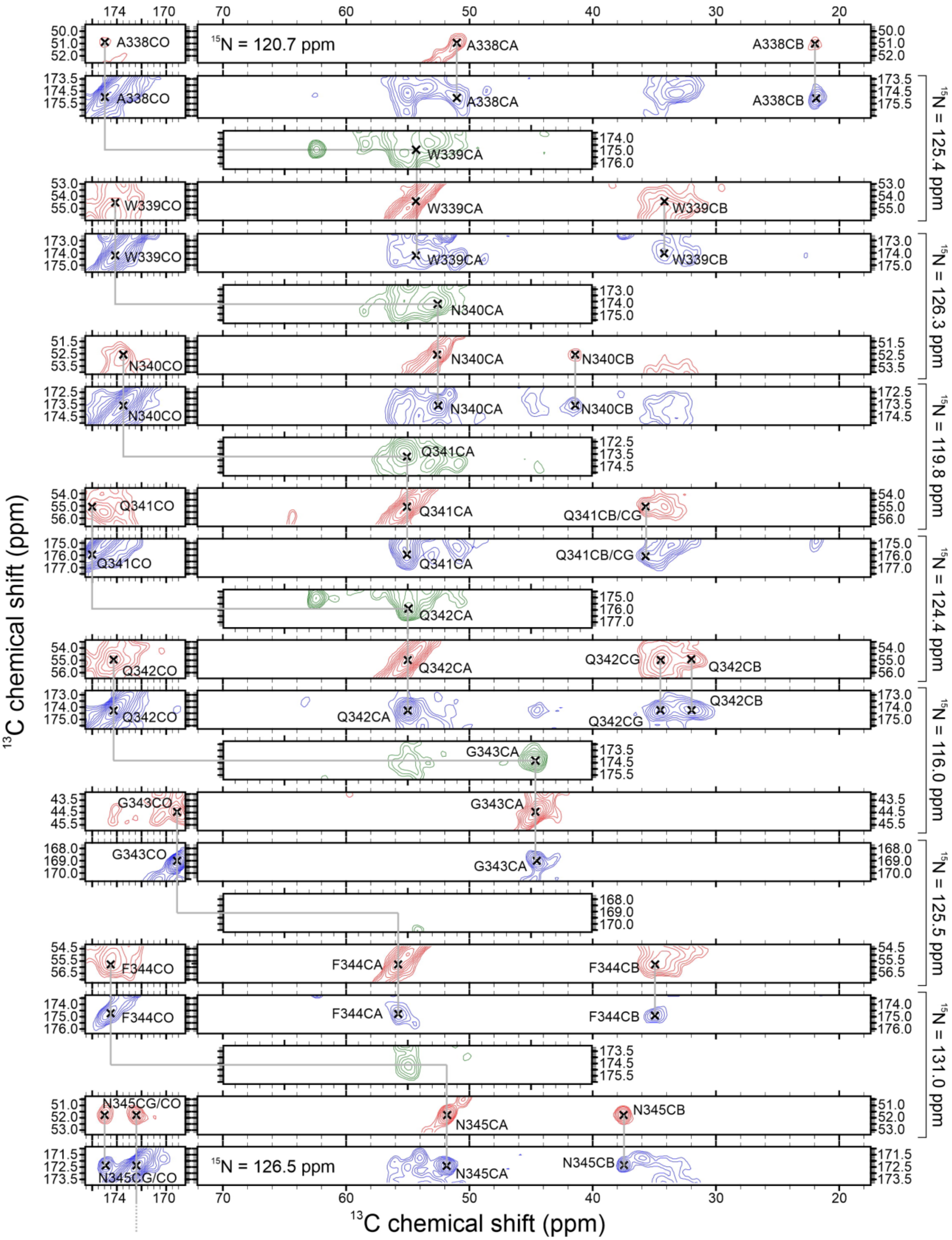

^{15}N = 120.7 ppm
A338CO
A338CA
A338CB
^{15}N = 125.4 ppm
W339CA
W339CO
W339CB
^{15}N = 126.3 ppm
N340CA
N340CO
N340CB
^{15}N = 119.8 ppm
Q341CA
Q341CO
Q341CB/CG
^{15}N = 124.4 ppm
Q342CA
Q342CO
Q342CG
Q342CB
^{15}N = 116.0 ppm
G343CA
G343CO
^{15}N = 125.5 ppm
F344CA
F344CO
F344CB
^{15}N = 131.0 ppm
N345CG/CO
N345CA
N345CB
^{15}N = 126.5 ppm
^{13}C chemical shift (ppm)

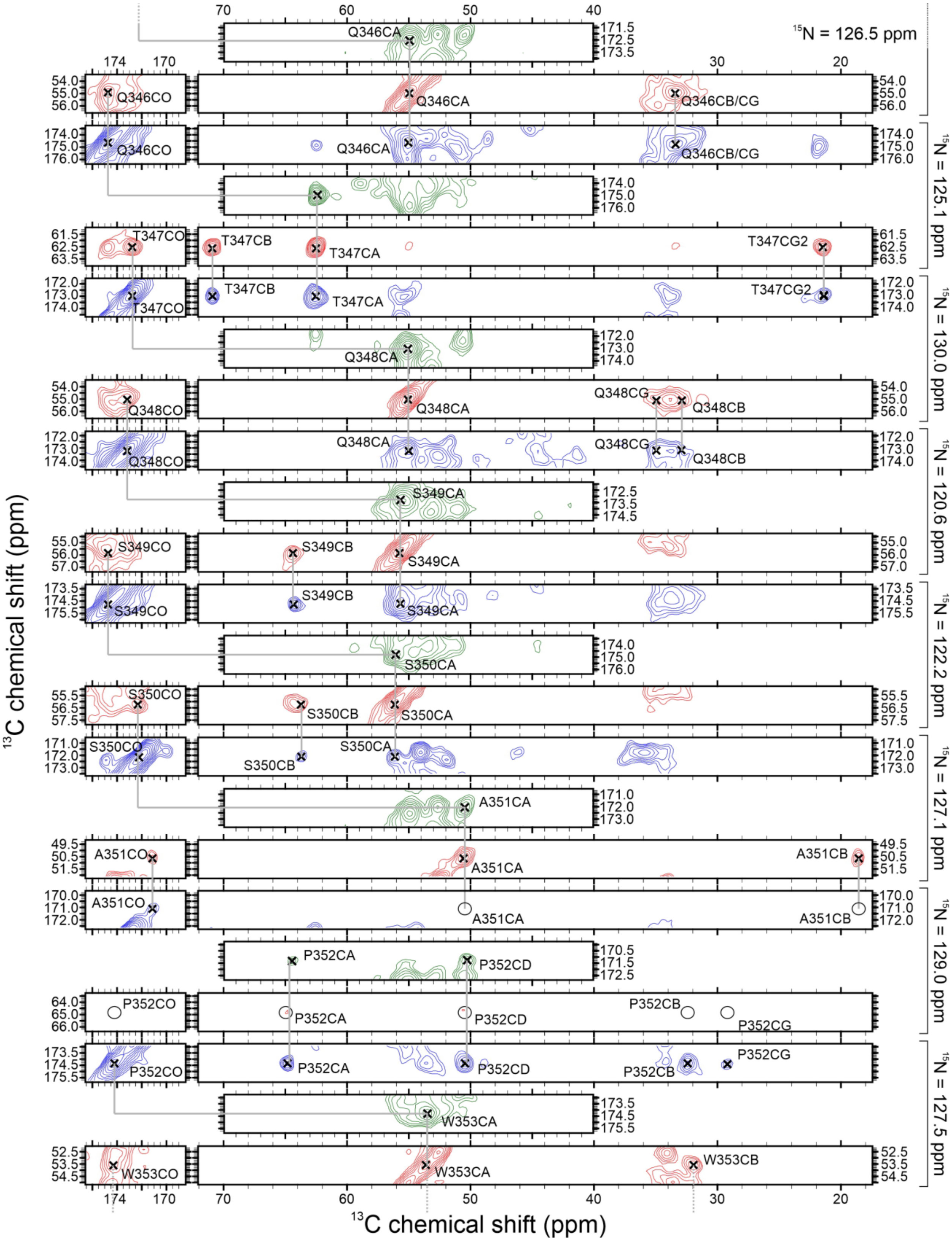

^{15}N = 126.5 ppm
^{15}N = 125.1 ppm
^{15}N = 130.0 ppm
^{15}N = 120.6 ppm
^{15}N = 122.2 ppm
^{15}N = 127.1 ppm
^{15}N = 129.0 ppm
^{15}N = 127.5 ppm
Q346CA
Q346CO
Q346CB/CG
T347CO
T347CB
T347CA
T347CG2
Q348CA
Q348CO
Q348CG
Q348CB
S349CA
S349CO
S349CB
S350CA
S350CO
S350CB
A351CA
A351CO
A351CB
P352CA
P352CD
P352CO
P352CB
P352CG
W353CA
W353CO
W353CB
^{13}C chemical shift (ppm)

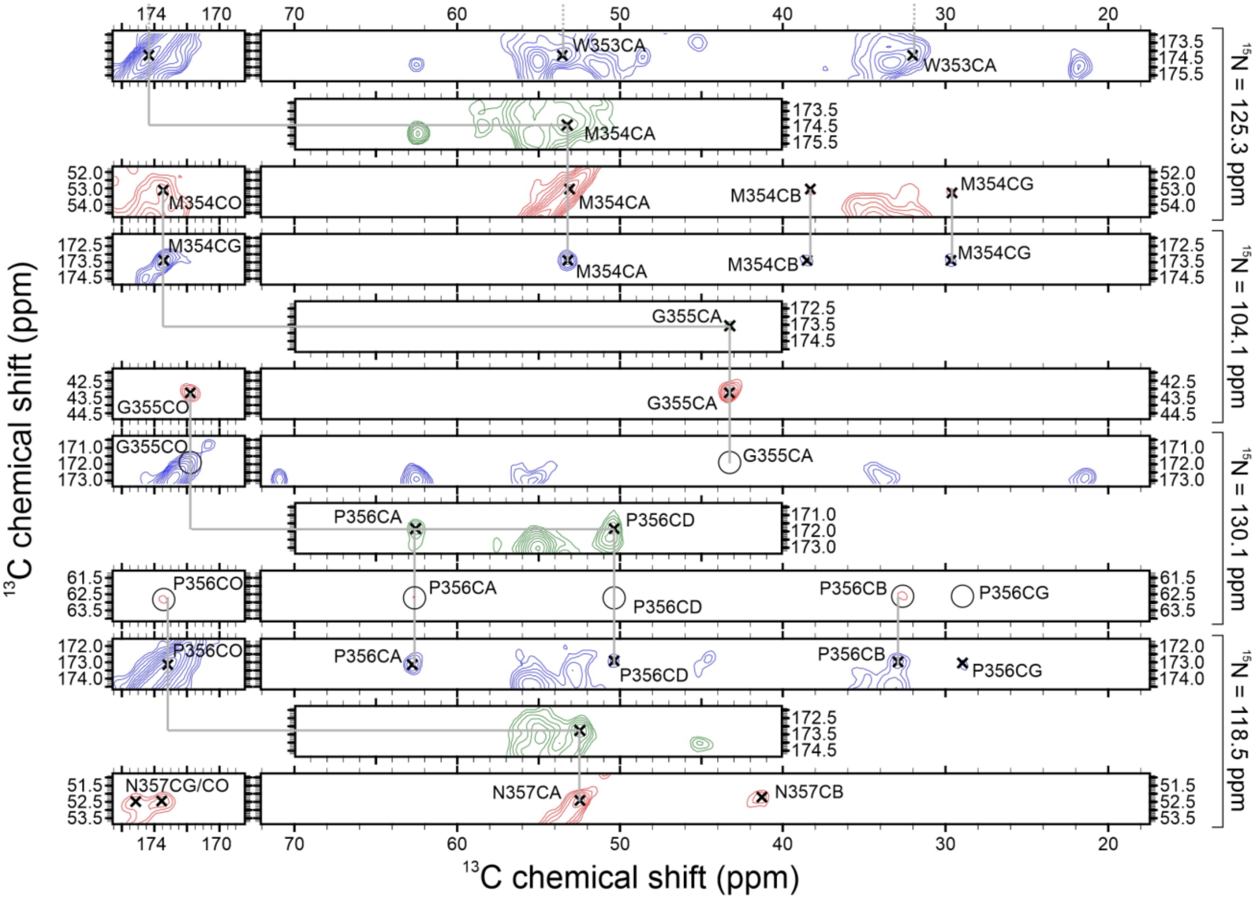

Supplemental Figure S5. Strip Plot of the Assigned Signals for the Seeded TIA1 LC Domain Fibrils. This figure spans three pages. 2D planes extracted from each 3D spectrum are displayed, with the NCACX colored red, the NCOCX colored blue, and the CANCO colored green. Gray lines trace the connections between each spectrum. Black circles indicate the positions of residues that are expected to be missing due to the presence of Pro resdiues.

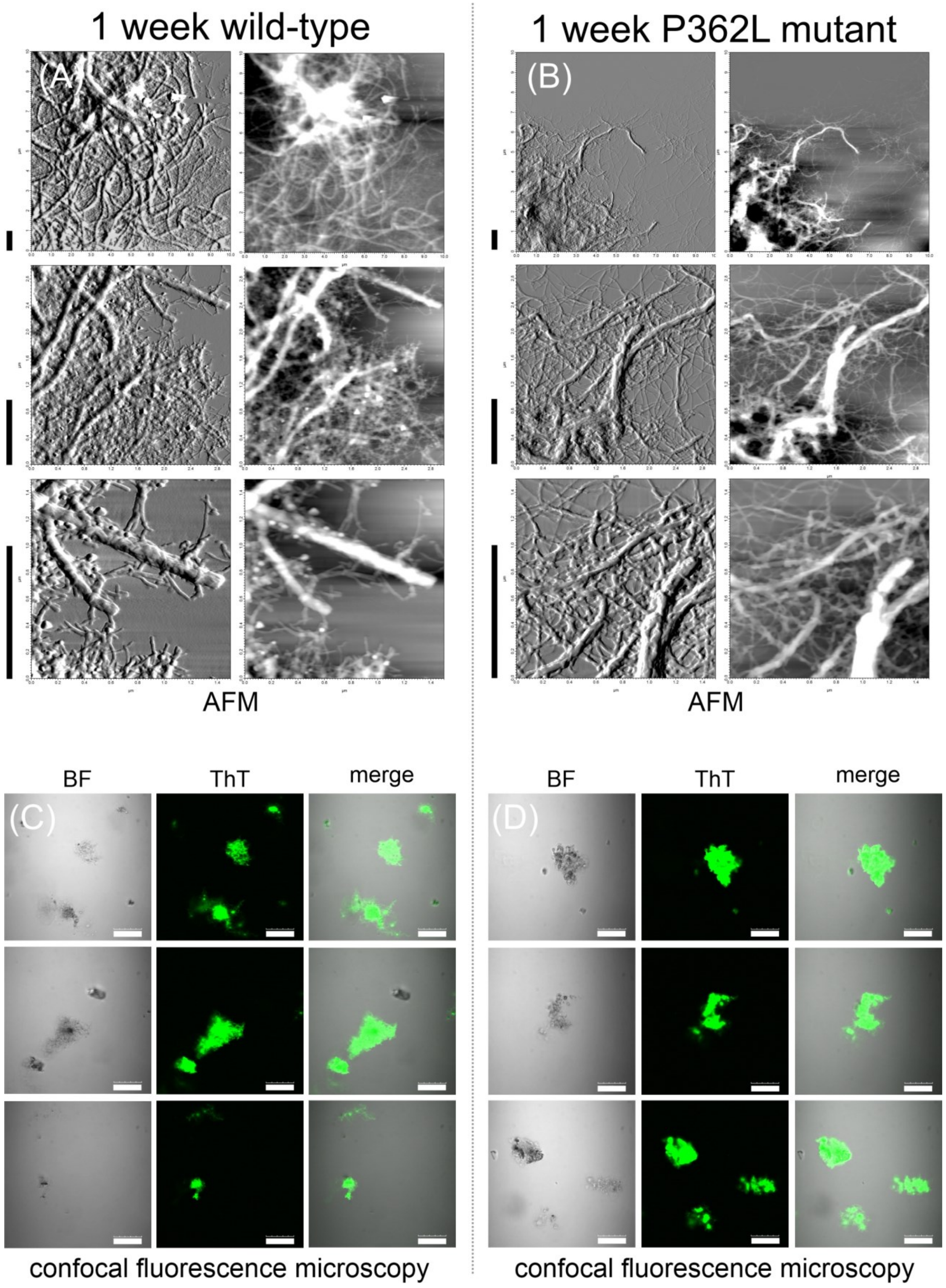
1 week wild-type
1 week P362L mutant
(A)
(B)
AFM
AFM
BF
ThT
merge
BF
ThT
merge
(C)
(D)
confocal fluorescence microscopy
confocal fluorescence microscopy

Supplemental Figure S6. Additional Analysis of TIA1 LC Domain Aged Liquid Droplets. (A) AFM images of wild-type TIA1 LC domain liquid droplets aged for 1 week. (B) AFM images of P362L mutant TIA1 LC domain liquid droplets aged for 1 week. Black vertical bars to the left of the images represent 1.0 μm. For both wild-type and P362L mutant, phase contrast images are in the left column and the corresponding height images are in the right column. (C) Confocal fluorescence microscopy images of wild-type TIA1 LC domain liquid droplets aged for 1 week. (D) Confocal fluorescence microscopy images of P362L mutant TIA1 LC domain liquid droplets aged for 1 week. The white bars represent 50 μm and the green intensity represents ThT fluorescence signal.

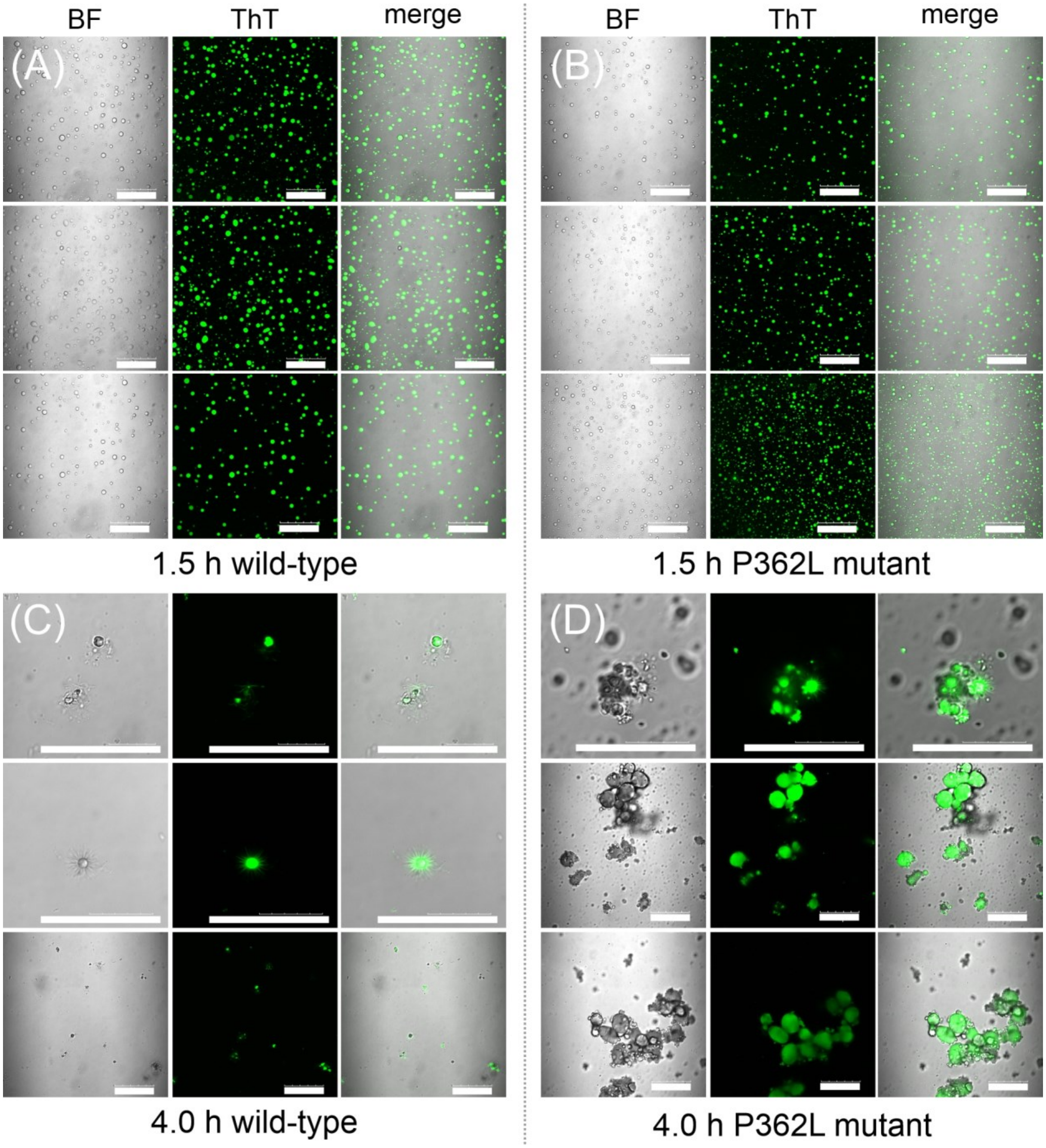

Supplemental Figure S7. Confocal Fluorescence Microscopy of TIA1 LC Domain Liquid Droplets at Short Incubation Times. (A) Wild-type TIA1 LC domain liquid droplets imaged at 1.5 h. (B) P362L mutant TIA1 LC domain liquid droplets imaged at 1.5 h. (C) Wild-type TIA1 LC domain liquid droplets imaged at 4.0 h. (D) P362L mutant TIA1 LC domain liquid droplets imaged at 4.0 h. The white bars represent 50 μm and the green intensity represents ThT fluorescence.

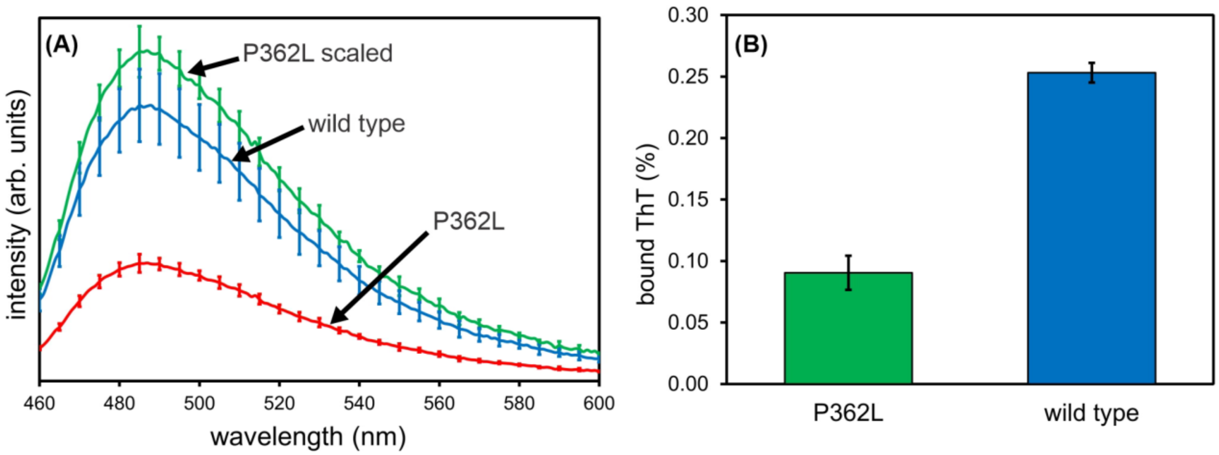

Supplemental Figure S8: Thioflavin-T Binding Assay. (A) Bulk ThT fluorescence spectra of TIA1 LC domain liquid droplets aged for 1 week. Red lines and error bars are for the P362L mutant sample and blue lines and error bars are for the wild-type sample. Green lines are the P362L mutant data scaled by the amount of bound ThT relative to the wild-type sample. (B) The percentage of ThT bound to the TIA1 LC domain in liquid droplet samples aged for 1 week, determined by absorbance measurements after centrifugation. In both (A) and (B), the error bars represent the standard deviation from three measurements.

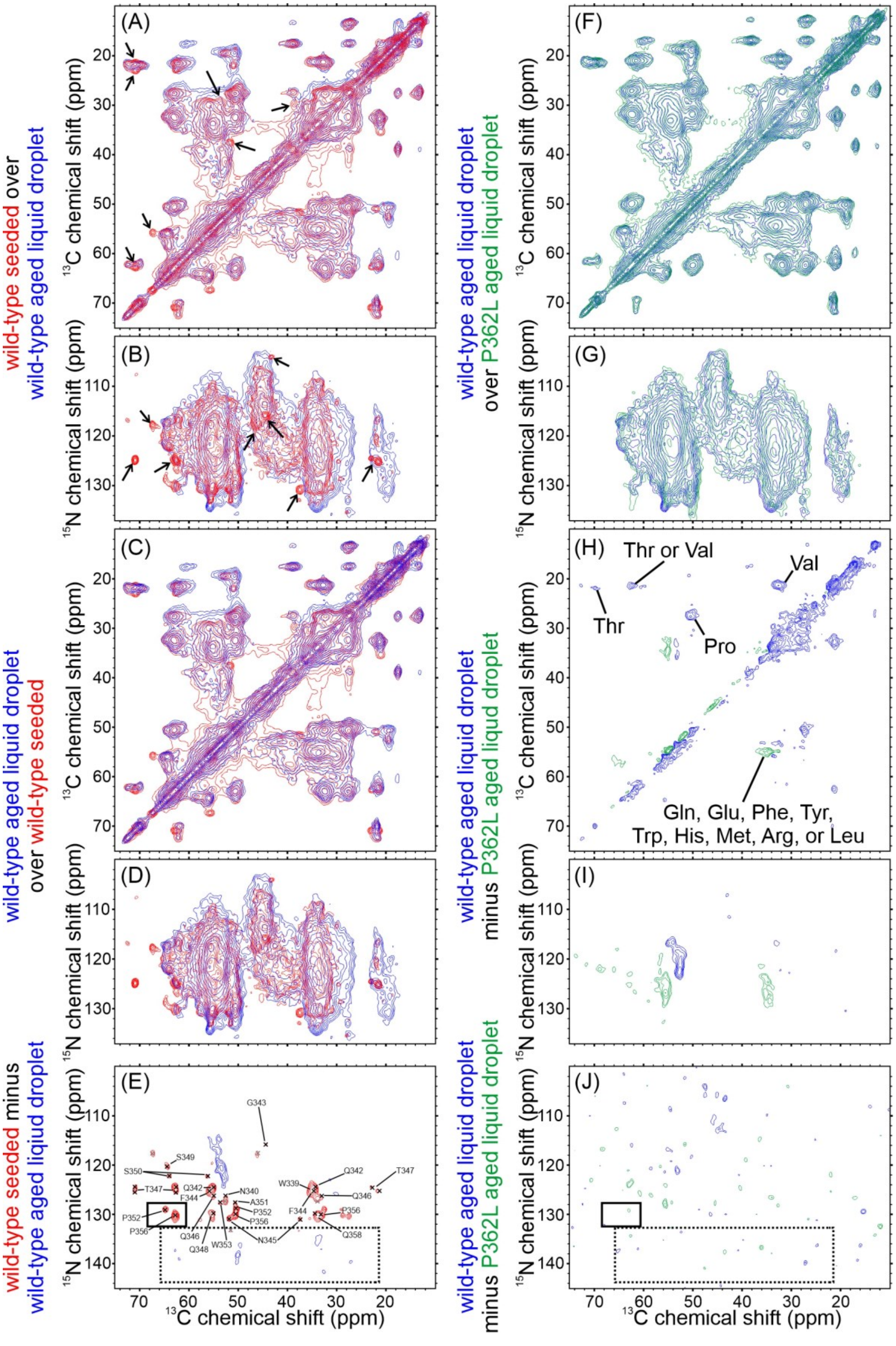
wild-type seeded over
wild-type aged liquid droplet
wild-type aged liquid droplet
over wild-type seeded
wild-type seeded minus
wild-type aged liquid droplet
wild-type aged liquid droplet
over P362L aged liquid droplet
wild-type aged liquid droplet
minus P362L aged liquid droplet
wild-type aged liquid droplet
minus P362L aged liquid droplet
(A)
(B)
(C)
(D)
(E)
(F)
(G)
(H)
(I)
(J)
13C chemical shift (ppm)
15N chemical shift (ppm)
Thr or Val
Val
Thr
Pro
Gln, Glu, Phe, Tyr,
Trp, His, Met, Arg, or Leu
G343
S349
S350
T347
Q342
N340
A351
F344
P352
P356
Q346
Q348
W353
N345
W339
Q358

Supplemental Figure S9. Comparison of Seeded Fibril and Aged Liquid Droplet Spectra for Wild-type and P362L Mutant TIA1 LC Domains. The label to the left of each spectrum indicates whether it is an overlay or difference spectrum and what two spectra were used to produce it, color coded to match the contours in the spectra. (A)–(D) overlays of the wild-type seeded and wild-type aged liquid droplet TIA1 LC domain 2D cross polarization-based spectra. Black arrows point to the locations of the most significant differences in (A) and (B). (E) difference spectrum for the TEDOR-based spectra of the wild-type seeded fibril and wild-type aged liquid droplet TIA1 LC domain samples. In (E) the blue contours represent signals that are more prominent in the aged liquid droplet sample, the red contours represent signals that are more prominent in the seeded fibril sample, the black solid box highlights the signals arising from conformationally homogenous and rigid Pro residues, and the black dashed box highlights the locations expected for the signals arising from the conformationally heterogenous but rigid Pro residues. (F) and (G) overlays of the aged liquid droplet cross polarization-based spectra from the wild-type and P362L mutant TIA1 LC domains. (H) and (I) the difference spectra calculated from the spectra in (F) and (G). The signals labeled in (H) were identified based on the statistical distributions of amino acid NMR chemical shifts in proteins. (J) the difference spectrum for the TEDOR-based spectra of the wild-type and P362L mutant aged liquid droplet TIA1 LC domain samples. In (H), (I), and (J) the green contours represent signals that are more prominent in the P362L mutant sample, and the blue contours represent signals that are more prominent in the wild-type sample. In (J) the black solid box highlights the signals arising from conformationally homogenous and rigid Pro residues, and the black dashed box highlights the locations expected for the signals arising from the conformationally heterogenous but rigid Pro residues. The contours in the carbon-carbon spectra are drawn at intensities increasing by a factor of 1.4. The contours in the nitrogen-carbon spectra are drawn at intensities increasing by a factor of 1.25.

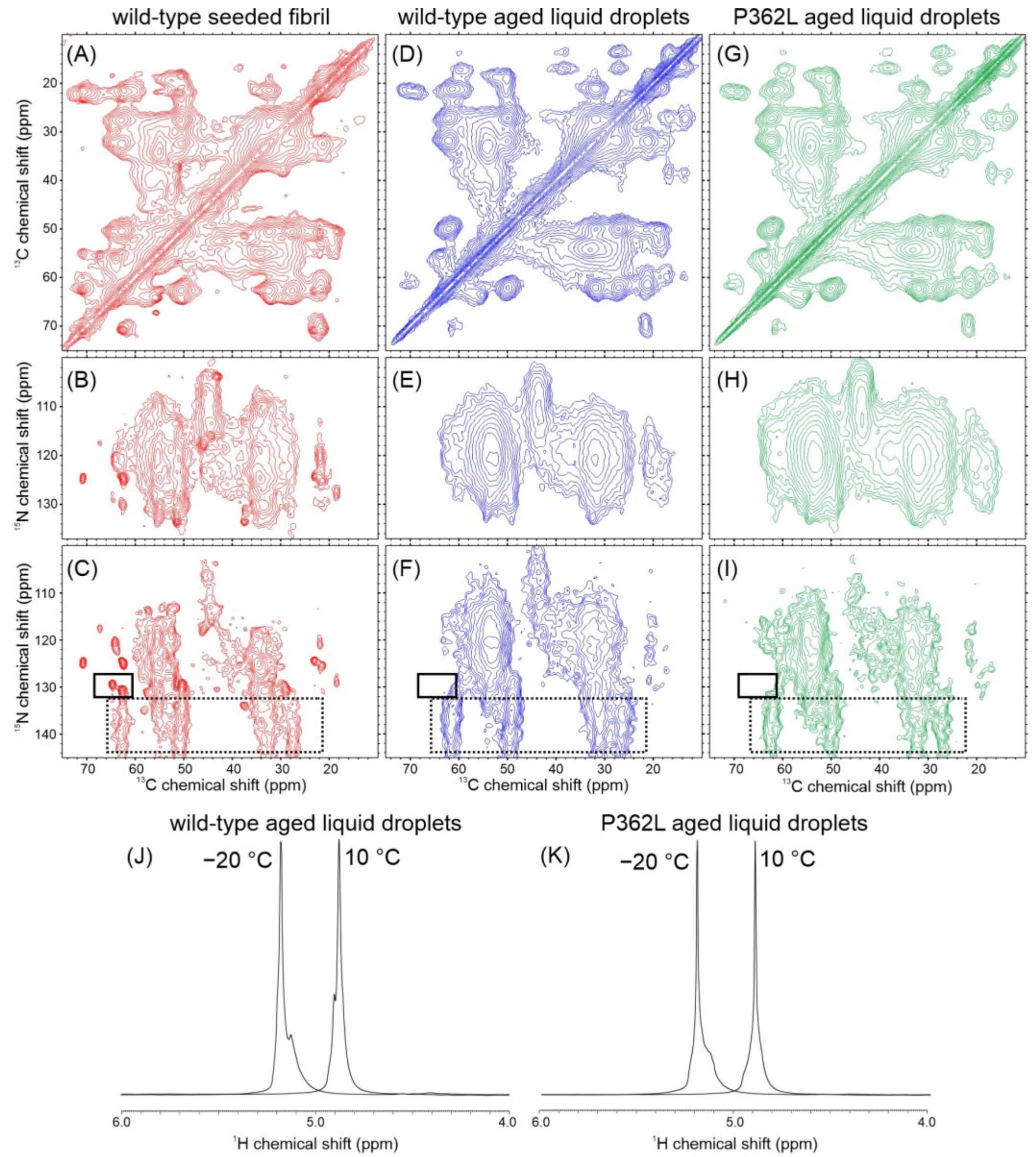

Supplemental Figure S10. Low Temperature Spectra of the TIA1 LC Domain Samples. The red contours are the wild-type TIA1 LC domain seeded fibrils. The blue contours are the wild-type TIA1 LC domain aged liquid droplets. The green contours are the P362L mutant TIA1 LC domain aged liquid droplets. (A), (D), and (G) are cross polarization-based carbon-carbon spectra with contours drawn at intensity levels increasing by a factor of 1.4. (B), (E), and (H) are cross polarization-based nitrogen-carbon spectra with contours increasing by a factor of 1.25. (C), (F), and (I) are TEDOR-based nitrogen-carbon spectra with contours increasing by a factor of 1.25. Dashed-line boxes are the signals arising from poorly ordered Pro residues. Solid-line boxes mark the locations expected for the well-ordered Pro residues present in the spectrum of the TIA1 LC domain wild-type seeded fibrils. The sample temperatures are approximately −20 °C. (J) Temperature dependent ^{1}H spectrum of wild-type TIA1 LC domain aged liquid droplets. (K) Temperature dependent ^{1}H spectrum of P362L mutant TIA1 LC domain aged liquid droplets.

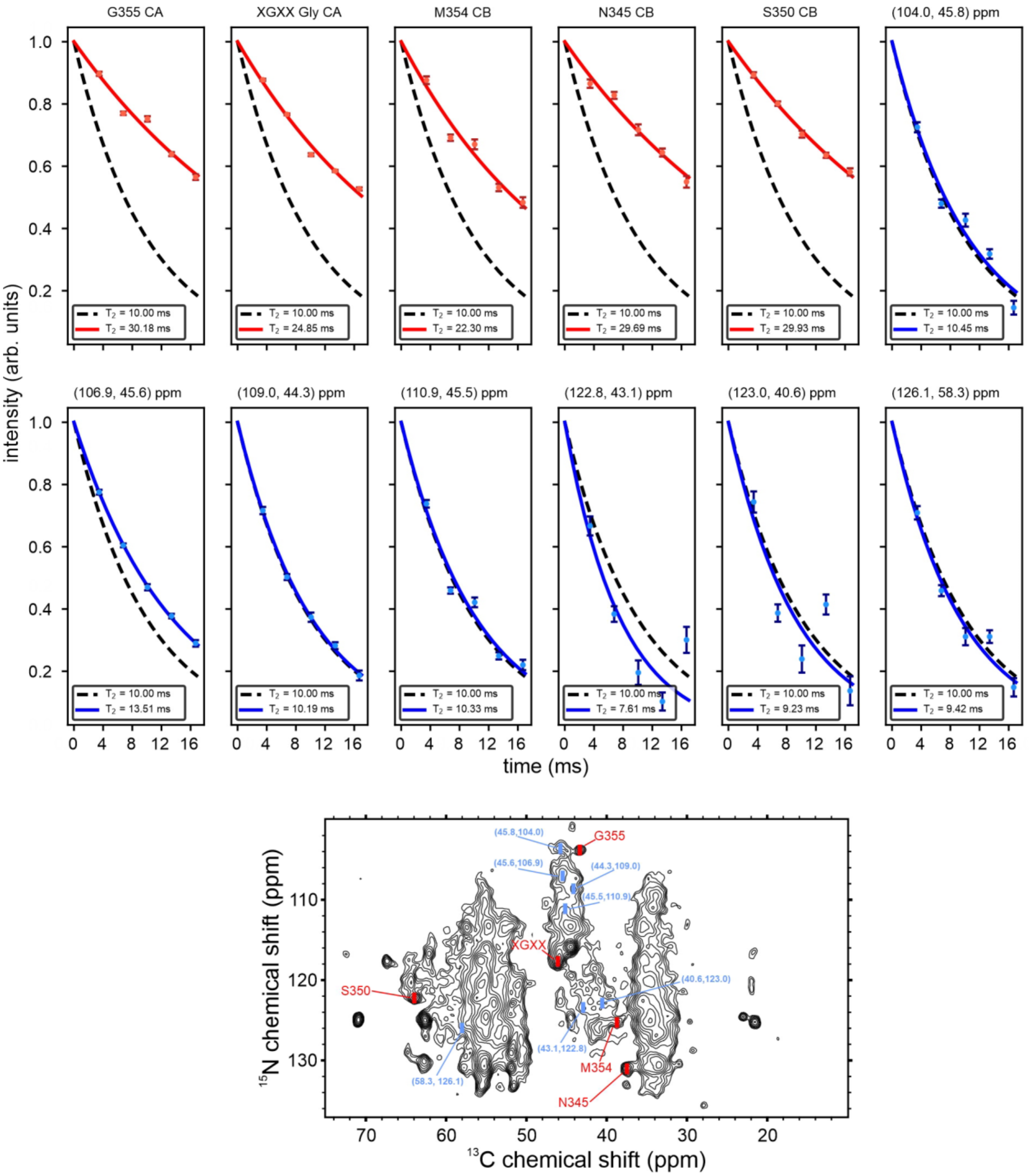

Supplemental Figure S11. Relaxation Measurements from 2D Cross Polarization-Based NCACX Spectra of Seeded TIA1 LC Domain Fibrils. Top panels show the decay curves used for measuring signal-specific ^{15}N T_2 values. Red curves and markers indicate strong, sharp signals and blue curves and markers indicate weaker, broad regions of signal intensities. Curves are fit to single-component exponential decays. A decay curve representing a T_2 of 10.0 ms is shown for reference in each panel. Integration boxes are +/− 0.2 ppm in the ^{13}C dimension and +/− 0.6 ppm in the ^{15}N dimension. The 2D cross-polarization NCACX spectrum from Figure 2B is shown for reference, with the integration boxes labeled.

Supplemental Table S1. Solid state NMR data collection and processing parameters.

	Spectrum	Acquisition Parameters[a,b]	Processing Parameters[c]
**Seeded Fibrils**	2D ^{13}C-^{13}C CP-DARR	ns=80; τ_{aq}=15.36 ms; $\nu_{1H\text{-}carr}$=3.4 ppm; $\nu_{13C\text{-}carr}$=98.0 ppm; $\nu_{1H\text{-}CP}$=63 kHz; $\nu_{13C\text{-}CP}$=50 kHz; ν_{1Hdec}=83.3 kHz; ν_{DARR}=13 kHz; ν_{MAS}=12.75 kHz; $\tau_{1H\text{-}\pi/2}$=3 μs; $\tau_{13C\text{-}\pi/2}$=4 μs; τ_{CP}=1.0 ms; τ_{DARR}=50 ms; $\tau_{1H\text{-}dec}$=4.9 μs; Δt_1=23.4 μs; τ_{t1}=5.5 ms; τ_{rec}=1.5 s;	GLB_{t1}=125 Hz;<br>GLB_{t2}=125 Hz;<br>LP in t_2
	2D CP-NCACX	ns=384; τ_{aq}=15.36 ms; $\nu_{1H\text{-}carr}$=3.4 ppm; $\nu_{13C\text{-}carr}$=55.0 ppm; $\nu_{15N\text{-}carr}$=118.0 ppm; $\nu_{1H\text{-}CP}$=46 kHz; $\nu_{15N\text{-}CP}$=33 kHz; $\nu_{15N\text{-}SCP}$=36 kHz; $\nu_{13CA\text{-}SCP}$=23 kHz; $\nu_{1H\text{-}dec}$=83.3 kHz; ν_{DARR}=13 kHz; ν_{MAS}=12.75 kHz; $\tau_{1H\text{-}\pi/2}$=3 μs; $\tau_{13C\text{-}\pi/2}$=4 μs; $\tau_{15N\text{-}\pi/2}$=6 μs; τ_{CP}=0.5 ms; τ_{SCP}=5.0 ms; τ_{DARR}=50 ms; $\tau_{1H\text{-}dec}$=4.9 μs; Δt_1=156.8 μs; τ_{t1}=11.6 ms; τ_{rec}=1.5 s;	GLB_{t1}=60 Hz;<br>GLB_{t2}=125 Hz;
	2D CP-NCOCX	ns=288; τ_{aq}=15.36 ms; $\nu_{1H\text{-}carr}$=3.4 ppm; $\nu_{13C\text{-}carr}$=176.8 ppm; $\nu_{15N\text{-}carr}$=118.0 ppm; $\nu_{1H\text{-}CP}$=46 kHz; $\nu_{15N\text{-}CP}$=33 kHz; $\nu_{15N\text{-}SCP}$=36 kHz; $\nu_{13CO\text{-}SCP}$=48 kHz; $\nu_{1H\text{-}dec}$=83.3 kHz; ν_{DARR}=13 kHz; ν_{MAS}=12.75 kHz; $\tau_{1H\text{-}\pi/2}$=3 μs; $\tau_{13C\text{-}\pi/2}$=4 μs; $\tau_{15N\text{-}\pi/2}$=6 μs; τ_{CP}=0.5 ms; τ_{SCP}=5.0 ms; τ_{DARR}=50 ms; $\tau_{1H\text{-}dec}$=4.9 μs; Δt_1=156.8 μs; τ_{t1}=11.6 ms; τ_{rec}=1.5 s;	GLB_{t1}=60 Hz;<br>GLB_{t2}=125 Hz;
	2D CP-TEDOR-NCACX	ns=800; τ_{aq}=15.36 ms; $\nu_{1H\text{-}carr}$=5.2 ppm; $\nu_{13C\text{-}carr}$=57.0 ppm; $\nu_{15N\text{-}carr}$=120.0 ppm; $\nu_{1H\text{-}CP}$=63 kHz; $\nu_{13C\text{-}CP}$=50 kHz; ν_{DARR}=13 kHz; ν_{1Hdec}=83.3 kHz; ν_{MAS}=12.7 kHz; $\tau_{1H\text{-}\pi/2}$=3 μs; $\tau_{13C\text{-}\pi/2}$=4 μs; $\tau_{13C\text{-}soft}$=431 μs; $\tau_{15N\text{-}\pi/2}$=6 μs; τ_{TEDOR}=1.6 ms; $\tau_{z\text{-}filter}$=78.4 μs; τ_{DARR}=50 ms; $\tau_{1H\text{-}dec}$=6.3 μs; Δt_1=156.9 μs; τ_{t1}=11.6 ms; τ_{rec}=1.5 s;	GLB_{t1}=60 Hz;<br>GLB_{t2}=125 Hz;
	2D ^{1}H-^{13}C INEPT	ns=16; τ_{aq}=20.48 ms; $\nu_{1H\text{-}carr}$=5.2 ppm; $\nu_{13C\text{-}carr}$=67.3 ppm; $\nu_{1H\text{-}dec}$=25.0 kHz; ν_{MAS}=12.75 kHz; $\tau_{1H\text{-}\pi/2}$=3 μs; $\tau_{13C\text{-}\pi/2}$=4 μs; $\tau_{J/2}$=1.2 ms; Δt_1=75.0 μs; τ_{t1}=11.25 ms; τ_{rec}=1.5 s;	GLB_{t1}=10 Hz;<br>GLB_{t2}=50 Hz;
	3D CP-NCACX	ns=16; τ_{aq}=10.24 ms; $\nu_{1H\text{-}carr}$=2.3 ppm; $\nu_{13C\text{-}carr}$=53.4 ppm; $\nu_{15N\text{-}carr}$=121.6 ppm; $\nu_{1H\text{-}CP}$=50 kHz; $\nu_{15N\text{-}CP}$=37 kHz; $\nu_{15N\text{-}SCP}$=33 kHz; $\nu_{13CA\text{-}SCP}$=20 kHz; $\nu_{1H\text{-}dec}$=83.3 kHz; ν_{DARR}=13 kHz; ν_{MAS}=12.65 kHz; $\tau_{1H\text{-}\pi/2}$=3 μs; $\tau_{13C\text{-}\pi/2}$=4 μs; $\tau_{15N\text{-}\pi/2}$=6 μs; τ_{CP}=0.5 ms; τ_{SCP}=5.0 ms; τ_{DARR}=50 ms; $\tau_{1H\text{-}dec}$=6.3 μs; Δt_2=88.2 μs; τ_{t2}=4.4 ms; Δt_1=138.6 μs; τ_{t1}=10.1 ms; τ_{rec}=1.5 s;	GLB_{t1}=60 Hz;<br>GLB_{t2}=125 Hz;<br>GLB_{t3}=125 Hz;

	3D CP-NCOCX	ns=32; τ_{aq}=10.24 ms; $\nu_{1H-carr}$=2.3 ppm; $\nu_{13C-carr}$=175.4 ppm; $\nu_{15N-carr}$=121.6 ppm; ν_{1H-CP}=50 kHz; ν_{15N-CP}=37 kHz; $\nu_{15N-SCP}$=33 kHz; $\nu_{13CO-SCP}$=46 kHz; ν_{1H-dec}=83.3 kHz; ν_{DARR}=13 kHz; ν_{MAS}=12.65 kHz; $\tau_{1H-\pi/2}$=3 µs; $\tau_{13C-\pi/2}$=4 µs; $\tau_{15N-\pi/2}$=6 µs; τ_{CP}=0.5 ms; τ_{SCP}=5.0 ms; τ_{DARR}=50 ms; τ_{1H-dec}=6.3 µs; Δt_2=138.6 µs; τ_{t2}=4.4 ms; Δt_1=138.6 µs; τ_{t1}=10.1 ms; τ_{rec}=1.5 s;	GLB_{t1}=60 Hz; GLB_{t2}=125 Hz; GLB_{t3}=125 Hz;
	3D CP-CANCO	ns=28; τ_{aq}=15.36 ms; $\nu_{1H-carr}$=1.0 ppm; $\nu_{13C-carr}$=55.0 ppm; $\nu_{15N-carr}$=118.0 ppm; ν_{1H-CP}=59 kHz; ν_{13C-CP}=46 kHz; $\nu_{15N-SCP}$=34 kHz; $\nu_{13CA-SCP}$=22 kHz; $\nu_{13CO-SCP-eff}$=47 kHz; ν_{1Hdec}=83.3 kHz; ν_{MAS}=12.7 kHz; $\tau_{1H-\pi/2}$=3 µs; τ_{SCP}=4 ms; τ_{1H-dec}=6 µs; Δt_2=151.2 µs; τ_{t2}=8.1 ms; Δt_1=75.6 µs; τ_{t1}=4.2 ms; τ_{rec}=1.5 s;	GLB_{t1}=125 Hz; GLB_{t2}=60 Hz; GLB_{t3}=125 Hz;
**Aged Droplet (WT and P36L Mutant)**	2D ^{13}C-^{13}C CP-DARR	$ns_{WT,263K}$=80; $ns_{WT,233K}$=96; $ns_{P362L,263K}$=64; $ns_{P362L,233K}$=80; τ_{aq}=7.68 ms; $\nu_{1H-carr}$=3.9 ppm; $\nu_{13C-carr}$=99.2 ppm; ν_{1H-CP}=63 kHz; ν_{13C-CP}=50 kHz; ν_{1Hdec}=83.3 kHz; ν_{DARR}=13 kHz; ν_{MAS}=12.75 kHz; $\tau_{1H-\pi/2}$=3 µs; $\tau_{13C-\pi/2}$=4 µs; τ_{CP}=1.0 ms; τ_{DARR}=50 ms; τ_{1H-dec}=6.0 µs; Δt_1=12.0 µs; $\tau_{t1,263K}$=3.9 ms; $\tau_{t1,233K}$=3.5 ms; τ_{rec}=2.0 s;	GLB_{t1}=75 Hz; GLB_{t2}=75 Hz; LP in t_2
	2D CP-NCACX	$ns_{WT,263K}$=616; $ns_{WT,233K}$=288; $ns_{P362L,263K}$=256; $ns_{P362L,233K}$=288; τ_{aq}=10.24 ms; $\nu_{1H-carr}$=3.9 ppm; $\nu_{13C-carr}$=63.8 ppm; $\nu_{15N-carr}$=120.4 ppm; ν_{1H-CP}=61 kHz; ν_{15N-CP}=49 kHz; $\nu_{15N-SCP}$=34 kHz; $\nu_{13CA-SCP}$=22 kHz; ν_{1H-dec}=83.3 kHz; ν_{DARR}=13 kHz; ν_{MAS}=12.75 kHz;$\tau_{1H-\pi/2}$=3 µs; $\tau_{13C-\pi/2}$=4 µs; $\tau_{15N-\pi/2}$=6 µs; τ_{CP}=0.75 ms; τ_{SCP}=4.0 ms; τ_{DARR}=50 ms; τ_{1H-dec}=6.0 µs; Δt_1=108.0 µs; $\tau_{t1,263K}$=10.0 ms; $\tau_{t1,233K}$=10.0 ms; τ_{rec}=2.0 s;	GLB_{t1}=80 Hz; GLB_{t2}=150 Hz;
	2D CP-TEDOR-NCACX	$ns_{WT,233K}$=512; $ns_{P362L,233K}$=576; τ_{aq}=7.68 ms; $\nu_{1H-carr}$=3.9 ppm; $\nu_{13C-carr}$=58.1 ppm; $\nu_{15N-carr}$=120.7 ppm; ν_{1H-CP}=63 kHz; ν_{13C-CP}=50 kHz; ν_{DARR}=13 kHz; ν_{1Hdec}=83.3 kHz; ν_{MAS}=12.75 kHz; $\tau_{1H-\pi/2}$=3 µs; $\tau_{13C-\pi/2}$=4 µs; $\tau_{13C-soft}$=431 µs; $\tau_{15N-\pi/2}$=6 µs; τ_{TEDOR}=1.6 ms; $\tau_{z-filter}$=78.4 µs; τ_{DARR}=50 ms; τ_{1H-dec}=6.0 µs; Δt_1=156.8 µs; τ_{t1}=8.3 ms; τ_{rec}=2.0 s;	GLB_{t1}=80 Hz; GLB_{t2}=150 Hz;

[a] All ^{1}H-X CP transfers used a 20% ramp on the ^{1}H channel. All ^{15}N-^{13}C Specific-CP transfers used a 5% ramp on the ^{15}N channel.

[b] ns, number of scans averaged (subscripts for the aged liquid droplet samples specify the sample and temperature); τ_{aq}, total acquisition time in the direct dimension; $\nu_{1H-carr}$, ^{1}H carrier frequency; $\nu_{13C-carr}$, ^{13}C carrier frequency; $\nu_{15N-carr}$, ^{15}N carrier frequency; ν_{1H-CP}, ^{1}H CP pulse power; ν_{13C-CP}, ^{13}C CP pulse power; ν_{15N-CP}, ^{15}N CP pulse power; $\nu_{15N-SCP}$, ^{15}N Specific-CP pulse power; ν_{13CA-}

$_{SCP}$, ^{13}C α-carbon Specific-CP pulse power; $\nu_{13CO\text{-}SCP}$, ^{13}C carbonyl-carbon Specific-CP pulse power; $\nu_{13CO\text{-}SCP\text{-}eff}$, ^{13}C carbonyl-carbon Specific-CP effective field pulse power; $\nu_{1H\text{-}dec}$, ^{1}H decoupling pulse power; ν_{DARR}, ^{13}C-^{13}C DARR ^{1}H pulse power; ν_{MAS}, magic angle spinning frequency; $\tau_{1H\text{-}\pi/2}$, ^{1}H π/2 pulse length; $\tau_{13C\text{-}\pi/2}$, ^{13}C π/2 pulse length; $\tau_{13C\text{-}soft}$, ^{13}C selective pulse length; $\tau_{15N\text{-}\pi/2}$, ^{15}N π/2 pulse length; τ_{CP}, ^{1}H-X CP contact time; τ_{SCP}, ^{15}N-^{13}C Specific-CP contact time; τ_{DARR}, ^{13}C-^{13}C DARR mixing time; $\tau_{J/2}$,1/2 echo delay; $\tau_{1H\text{-}dec}$, ^{1}H SPINAL64 π pulse length; Δt_2, time increment in the t_2 dimension; τ_{t2}, total acquisition time in the t_2 dimension; Δt_1, time increment in the t_1 dimension; τ_{t1}, total acquisition time in the t_1 dimension (subscripts for the aged liquid droplet samples specify the temperature); τ_{rec}, recycle delay; $\tau_{z\text{-}filter}$, z-filter time for the TEDOR experiments; τ_{TEDOR} total TEDOR mixing time;

[c] Zero filling was applied twice in each dimension prior to Fourier transformation. GLB, gaussian line broadening and LP, forward linear prediction with standard NMRPipe parameters.

Supplemental Table S2. Assigned solid state NMR chemical shifts (ppm). Signals in the table represent the average from the NCACX, NCOCX, and CANCO 3D spectra.

**Unambiguously Assigned Chemical Shifts**						
Residue	N	CA	C	CB	CG	CD
Q337			174.0			
A338	120.7	51.0	175.0	21.9		
W339	125.4	54.0	174.3	34.2		
N340	126.3	52.6	173.7	41.5		
Q341	119.8	55.1	175.9	35.4	35.4	179.2
Q342	124.4	54.9	174.3	32.0	34.5	177.0
G343	116.0	44.5	169.1			
F344	125.5	55.8	174.6	34.9		
N345	131.0	51.9	172.5	37.5	174.9	
Q346	126.5	55.0	174.7	33.3	33.2	178.4
T347	125.1	62.5	173.5	71.0	21.5/23.0[a]	
Q348	130.0	55.0	173.3	33.2	34.8	
S349	120.6	55.8	174.8	64.3		
S350	122.2	56.2	172.1	63.9		
A351	127.1	50.6	171.2	18.6		
P352	129.0	64.7	174.3	32.5	29.1	50.3
W353	127.5	53.6	174.3	32.0		
M354	125.3	53.2	173.5	38.4	29.7	
G355	104.1	43.3	171.8			
P356	130.1	62.7	173.4	32.9	28.6	50.5
N357	118.5	52.4	173.5	41.3	175.2	
**XGXX Motif (ppm)**						
X			176.1			
G	117.8	46.0	169.9			
X	122.9	54.0	171.9	35.8		
X	127.3	52.6	174.0	34.1		

[a]Two Thr signals are present with nearly identical CA and CB chemical shifts but differing CG2 chemical shifts.

Supplemental Table S3. NCACX MCASSIGN Table. Chemical shifts and uncertainties are in ppm. Amino acid types are single letter codes. A value of '1111.1' is used to indicate no signal.

N	CA	CO	CB	CG	CD	ΔN	ΔCA	ΔCO	ΔCB	ΔCG	ΔCD	Amino Acid Type
104.2	43.2	171.8	1111.1	1111.1	1111.1	0.4	0.3	0.3	0.3	0.3	0.3	G
113.8	53.0	174	44.1	177.1	1111.1	0.4	0.4	0.4	0.4	0.4	0.3	ND
116.0	44.4	169.2	1111.1	1111.1	1111.1	0.4	0.4	0.6	0.3	0.3	0.3	G
117.7	45.9	169.8	1111.1	1111.1	1111.1	0.4	0.4	0.6	0.3	0.3	0.3	G
117.7	55.7	174.2	67.2	1111.1	1111.1	0.4	0.3	0.8	0.3	0.3	0.3	S
117.8	55.8	174.3	64.7	1111.1	1111.1	0.4	0.3	0.8	0.3	0.3	0.3	S
118.6	52.4	173.7	41.4	175.2	1111.1	0.4	0.3	0.5	0.3	0.6	0.3	ND
119.3	55.2	175	34.3	1111.1	1111.1	0.5	0.3	1.5	0.5	1.2	0.5	RDNQEHILMFWY
119.6	55.1	175.8	35.5	1111.1	1111.1	0.4	0.3	1.5	0.4	0.3	0.3	RDNQEHILMFWY
120.6	55.8	174.2	64.3	1111.1	1111.1	0.4	0.3	1.1	0.3	0.3	0.3	S
120.7	50.9	175	21.8	1111.1	1111.1	0.4	0.3	0.5	0.3	0.3	0.3	A
120.8	55.4	174.5	33.8	1111.1	1111.1	0.5	0.4	1.5	0.8	0.3	0.3	RDNQEHILMFWY
122.3	56.2	172.3	63.9	1111.1	1111.1	0.4	0.3	0.3	0.3	0.3	0.3	S
122.4	55.3	174.4	33.9	1111.1	1111.1	0.4	0.5	0.5	1.5	0.3	0.3	RDNQEHILMFWY
122.9	54.0	171.8	35.7	1111.1	1111.1	0.4	0.3	0.3	0.7	0.3	0.3	RDNQEHILMFWY
124.5	54.9	174.3	31.9	34.3	176.9	0.5	0.4	0.4	0.7	0.7	0.7	QE
124.5	62.6	172.8	70.9	23.0	1111.1	0.4	0.3	0.3	0.3	0.3	0.3	T
124.5	54.9	174.2	34.5	1111.1	1111.1	0.5	0.4	0.8	0.7	0.7	0.8	RDNQEHILMFWY
125.1	53.1	173.6	38.5	29.5	1111.1	0.4	0.3	1.0	0.3	0.3	0.3	RDNQEHILMFWY
125.2	55.8	174.3	34.7	1111.1	1111.1	0.5	0.4	0.7	0.7	0.3	0.3	RDNQEHILMFWY
125.3	62.6	172.8	70.9	21.4	1111.1	0.4	0.3	0.3	0.3	0.3	0.3	T
125.4	54.0	173.1	34.8	1111.1	1111.1	0.6	0.4	1.2	1.0	0.3	0.3	RDNQEHILMFWY
126.2	52.4	173.6	41.4	1111.1	1111.1	0.4	0.3	0.4	0.3	0.3	0.3	RDNQEHILMFWY
126.5	55.0	174.7	33.2	33.2	178.4	0.4	0.3	0.3	1.6	1.6	0.3	QE
127.3	52.6	174	34.1	1111.1	1111.1	0.4	0.3	0.4	0.3	0.3	0.3	RDNQEHILMFWY
127.4	50.5	171.2	18.5	1111.1	1111.1	0.4	0.3	0.3	0.3	0.3	0.3	A
127.5	53.7	174.1	31.9	1111.1	1111.1	0.4	0.3	0.7	0.3	0.3	0.3	RDNQEHILMFWY
129.1	64.7	174.2	32.5	29.0	50.2	0.4	0.3	0.7	0.3	0.3	0.3	P
129.8	55.0	173.2	32.7	34.5	1111.1	0.4	0.3	0.4	0.7	0.7	0.3	RDNQEHILMFWY
130.3	62.8	173.7	32.9	28.2	50.6	0.4	0.3	1.7	1.4	0.9	0.4	P
130.9	51.8	172.4	37.4	175.0	1111.1	0.4	0.3	0.3	0.3	0.3	0.3	ND
133.0	55.5	173.9	34.0	1111.1	1111.1	0.5	0.4	0.4	0.7	0.3	0.3	RDNQEHILMFWY
133.2	51.4	172.5	37.6	175.8	1111.1	0.4	0.3	0.3	0.3	0.3	0.3	ND

Supplemental Table S4. NCOCX MCASSIGN Table. Chemical shifts and uncertainties are in ppm. Amino acid types are single letter codes. A value of '1111.1' is used to indicate no signal.

N	CA	CO	CB	CG	CD	ΔN	ΔCA	ΔCO	ΔCB	ΔCG	ΔCD	Amino Acid Type
104.0	53.2	173.4	38.5	29.7	1111	0.3	0.3	0.3	0.3	0.3	0.4	RDNQEHILMFWY
113.6	43.9	171.7	1111.1	1111.1	1111.1	0.3	0.3	0.3	0.4	0.4	0.4	G
116.0	55.0	174.3	32.0	34.4	177.1	0.3	0.3	0.3	0.5	0.3	0.4	QE
117.5	55.3	174.6	33.1	1111.1	1111.1	0.4	0.3	0.3	0.3	0.4	0.4	RDNQEHILMFWY
118.3	62.8	173.1	32.9	29.0	50.4	0.4	0.4	0.4	0.4	0.4	0.4	P
119.5	55.6	174.7	34.1	1111.1	1111.1	0.5	0.4	0.4	2.0	0.4	0.4	RDNQEHILMFWY
119.7	52.5	173.6	41.5	1111.1	1111.1	0.4	0.3	0.3	0.3	0.4	0.4	RDNQEHILMFWY
120.7	55.0	173.3	33.9	1111.1	1111.1	0.4	0.4	0.3	1.5	0.4	0.4	RDNQEHILMFWY
121.0	52.7	173.6	33.8	1111.1	1111.1	0.4	0.3	0.3	1.5	0.4	0.4	RDNQEHILMFWY
122.2	44.4	172.4	1111.1	1111.1	1111.1	0.4	0.7	0.7	0.4	0.4	0.4	G
122.3	55.7	174.8	64.2	1111.1	1111.1	0.3	0.3	0.3	0.3	0.4	0.4	S
122.3	55.0	174	33.7	1111.1	1111.1	0.5	0.7	1.0	2.0	0.4	0.4	RDNQEHILMFWY
122.9	46.0	169.9	1111.1	1111.1	1111.1	0.3	0.6	0.6	0.4	0.4	0.4	G
123.5	52.8	172.6	1111.1	1111.1	1111.1	0.4	0.8	0.4	0.4	0.4	0.4	RDNQEHILMFWYS
123.6	53.7	173.8	33.6	1111.1	1111.1	0.4	0.4	0.4	2.0	0.4	0.4	RDNQEHILMFWY
123.7	55.3	174.1	33.6	1111.1	1111.1	0.4	0.4	0.4	2.0	0.4	0.4	RDNQEHILMFWY
124.3	55.1	176.3	35.1	1111.1	1111.1	0.4	0.3	0.4	0.5	0.4	0.4	RDNQEHILMFWY
124.9	55.1	174.7	33.3	1111.1	1111.1	0.4	0.4	0.4	1.5	0.4	0.4	RDNQEHILMFWY
125.2	53.6	174.3	32.5	1111.1	1111.1	0.4	0.4	0.4	1.0	0.4	0.4	RDNQEHILMFWY
125.4	51.0	175.1	21.8	1111.1	1111.1	0.4	0.3	0.3	0.3	0.4	0.4	A
125.6	44.5	169.2	1111.1	1111.1	1111.1	0.3	0.3	0.5	0.4	0.4	0.4	G
126.1	54.9	172.6	33.4	1111.1	1111.1	0.4	0.4	0.4	0.8	0.4	0.4	RDNQEHILMFWY
126.4	53.6	174.3	34.1	1111.1	1111.1	0.4	0.8	0.4	0.8	0.4	0.4	RDNQEHILMFWY
126.5	51.8	172.3	37.5	174.8	1111.1	0.3	0.3	0.3	0.3	0.3	0.4	ND
126.5	50.9	174.4	32.3	1111.1	1111.1	0.4	0.4	0.4	0.4	0.4	0.4	RDNQEHILMFWY
127.2	56.1	172.1	63.7	1111.1	1111.1	0.4	0.4	0.4	0.3	0.4	0.4	S
127.3	54.0	171.9	35.6	1111.1	1111.1	0.3	0.3	0.3	1.5	0.4	0.4	RDNQEHILMFWY
127.4	64.8	174.3	32.4	29.2	50.3	0.3	0.3	0.3	0.3	0.3	0.3	P
128.5	52.7	174.2	34.3	1111.1	1111.1	0.4	0.3	0.3	0.3	0.4	0.4	RDNQEHILMFWY
128.7	56.3	173.0	1111.1	1111.1	1111.1	0.4	0.3	0.3	0.4	0.4	0.4	RDNQEHILMFWYS
129.4	62.7	173.0	70.9	23.0	1111.1	0.4	0.3	0.3	0.3	0.3	0.3	T
129.7	55.7	174.8	34.4	1111.1	1111.1	0.4	0.5	0.4	1.0	0.4	0.4	RDNQEHILMFWY
129.7	55.3	173.0	33.9	1111.1	1111.1	0.4	0.7	0.4	0.7	0.4	0.4	RDNQEHILMFWY
130.3	62.5	172.8	71.0	21.4	1111.1	0.5	0.3	0.3	0.3	0.3	0.4	T
130.7	56.3	172.6	34.0	1111.1	1111.1	0.4	0.5	0.4	0.5	0.4	0.4	RDNQEHILMFWY
131.0	55.7	175.0	34.9	1111.1	1111.1	0.4	0.3	0.3	0.3	0.4	0.4	RDNQEHILMFWY
133.4	53.3	172.3	1111.1	1111.1	1111.1	0.4	0.4	0.4	0.4	0.4	0.4	RDNQEHILMFWYS

Supplemental Table S5. CANCO MCASSIGN Table. Chemical shifts and uncertainties are in ppm. Amino acid types are single letter codes. A value of '1111.1' is used to indicate no signal.

N	CA	CO	CB	CG	CD	ΔN	ΔCA	ΔCO	ΔCB	ΔCG	ΔCD	Amino Acid Type
104.2	43.3	173.6	1111.1	1111.1	1111.1	0.4	0.7	0.4	0.4	0.4	0.4	G
115.9	44.6	174.3	1111.1	1111.1	1111.1	0.4	0.3	0.3	0.4	0.4	0.4	G
117.5	55.6	174.6	1111.1	1111.1	1111.1	0.4	0.3	0.3	0.4	0.4	0.4	S
117.6	45.9	176.3	1111.1	1111.1	1111.1	0.4	0.3	0.3	0.4	0.4	0.4	G
118.5	52.5	173.3	1111.1	1111.1	1111.1	0.4	0.3	0.3	0.4	0.4	0.4	RDNQEHILMFWY
119.5	55.1	174.0	1111.1	1111.1	1111.1	0.4	0.4	2.0	0.4	0.4	0.4	RDNQEHILMFWY
120.5	55.9	173.3	1111.1	1111.1	1111.1	0.4	0.4	0.4	0.4	0.4	0.4	S
120.7	53.2	174.3	1111.1	1111.1	1111.1	0.4	0.3	0.3	0.4	0.4	0.4	RDNQEHILMFWY
120.8	51.0	174.1	1111.1	1111.1	1111.1	0.4	0.3	0.3	0.4	0.4	0.4	A
121.4	53.2	173.5	1111.1	1111.1	1111.1	0.4	0.4	0.7	0.4	0.4	0.4	RDNQEHILMFWY
122.0	54.6	174.6	1111.1	1111.1	1111.1	0.4	0.4	0.4	0.4	0.4	0.4	RDNQEHILMFWY
122.2	56.1	174.8	1111.1	1111.1	1111.1	0.4	0.4	0.4	0.4	0.4	0.4	S
122.3	55.4	173.6	1111.1	1111.1	1111.1	0.8	0.8	1.5	0.4	0.4	0.4	RDNQEHILMFWY
122.8	51.1	174.2	1111.1	1111.1	1111.1	0.4	0.3	0.3	0.4	0.4	0.4	RDNQEHILMFWY
122.9	54.0	170.0	1111.1	1111.1	1111.1	0.4	0.3	1.0	0.4	0.4	0.4	RDNQEHILMFWY
123.0	53.4	174.0	1111.1	1111.1	1111.1	0.4	0.3	0.4	0.4	0.4	0.4	RDNQEHILMFWY
124.3	54.9	175.9	1111.1	1111.1	1111.1	0.4	0.3	0.3	0.4	0.4	0.4	RDNQEHILMFWY
124.3	55.1	174.2	1111.1	1111.1	1111.1	0.5	0.5	2.0	0.4	0.4	0.4	RDNQEHILMFWY
124.8	62.4	175.0	1111.1	1111.1	1111.1	0.8	0.4	0.4	0.4	0.4	0.4	T
124.9	54.0	170.6	1111.1	1111.1	1111.1	0.4	0.3	1.0	0.4	0.4	0.4	RDNQEHILMFWY
125.4	54.0	174.9	1111.1	1111.1	1111.1	0.4	0.4	0.4	0.4	0.4	0.4	RDNQEHILMFWY
125.4	53.3	174.4	1111.1	1111.1	1111.1	0.4	0.4	0.4	0.4	0.4	0.4	RDNQEHILMFWY
126.2	52.7	173.9	1111.1	1111.1	1111.1	0.4	0.5	1.0	0.4	0.4	0.4	RDNQEHILMFWY
126.4	54.9	172.6	1111.1	1111.1	1111.1	0.4	0.3	0.3	0.4	0.4	0.4	RDNQEHILMFWY
126.4	55.3	174.0	1111.1	1111.1	1111.1	0.4	0.4	0.8	0.4	0.4	0.4	RDNQEHILMFWY
126.8	50.5	172.1	1111.1	1111.1	1111.1	0.6	0.4	0.4	0.4	0.4	0.4	A
127.1	52.7	172.0	1111.1	1111.1	1111.1	0.4	0.3	0.3	0.4	0.4	0.4	RDNQEHILMFWY
127.5	53.5	174.3	1111.1	1111.1	1111.1	0.4	0.4	0.4	0.4	0.4	0.4	RDNQEHILMFWY
128.8	64.5	171.3	1111.1	1111.1	50.3	0.4	0.4	0.4	0.4	0.4	0.4	P
129.4	50.6	172.4	1111.1	1111.1	1111.1	0.6	0.4	0.8	0.4	0.4	0.4	A
129.7	55.1	173.0	1111.1	1111.1	1111.1	0.4	0.3	0.3	0.4	0.4	0.4	RDNQEHILMFWY
129.9	62.6	171.8	1111.1	1111.1	1111.1	0.4	0.3	0.3	0.4	0.4	0.4	P
130.4	55.0	174.7	1111.1	1111.1	1111.1	0.4	0.3	0.3	0.4	0.4	0.4	RDNQEHILMFWY
133.1	55.5	173.8	1111.1	1111.1	1111.1	0.4	0.4	0.8	0.4	0.4	0.4	RDNQEHILMFWY
